# Global burden of stroke attributable to dietary risk factors in the GBD 2021 study

**DOI:** 10.3389/fnut.2024.1494574

**Published:** 2024-12-30

**Authors:** Wen Xie, Aiping Li, Yao Zhang, Shenglan Tan, Juan Luo

**Affiliations:** ^1^Department of Neurology, Hunan Provincial People's Hospital, Changsha, Hunan, China; ^2^Department of Pharmacy, Second Xiangya Hospital, Central South University, Changsha, Hunan, China; ^3^Department of Endocrine, Hunan Provincial People's Hospital, Changsha, Hunan, China

**Keywords:** stroke, dietary risk factors, age-standardized death rate, disability-adjusted life years, global burden of disease

## Abstract

**Objective:**

We sought to assess the impact of dietary risk on the worldwide burden of stroke, focusing specifically on ischemic stroke.

**Methods:**

Utilizing information from the Global Burden of Disease Study 2021 (GBD2021), we evaluated the age-standardized death rate (ASDR), the age-standardized disability-adjusted life years (DALYs) rate, and the age, sex, and regional distribution of the estimated annual percentage change (EAPC) of the stroke burden linked to dietary risk from 1990 to 2021.

**Results:**

The global overall ASDR and the age-standardized DALY rate per 100,000 population for stroke linked to dietary risk from 1990 to 2021 exhibited a declining trend [EAPC = −1.95; EAPC = −1.70, respectively]. The reduction in ASDR was statistically more pronounced in female (EAPC = −2.42) compared to males (EAPC = −1.60). The dietary factor exerting the most significant impact on stroke in 2021 was a high sodium diet, succeeded by a diet deficient in fruit. The regions and countries most affected by a high-sodium diet on the ASDR for ischemic stroke are Central Europe (9.86 per 100,000 population) and North Macedonia (33.13 per 100,000 population), respectively; the regions and countries with the most substantial influence on the age-standardized DALY rate are East Asia (187.15 per 100,000 population) and North Macedonia (477.26 per 100,000 population). The ASDR and age-standardized DALY rates across 5 Socio-Demographic Index (SDI) regions, 20 regions, and over 170 countries worldwide demonstrated a notable downward trend, with the regions experiencing the most significant decline being High SDI (EAPC: −3.64; EAPC: −2.74, respectively). The sole increase in ASDR was recorded in southern sub-Saharan Africa.

**Conclusion:**

The worldwide toll of stroke linked to dietary risks may have diminished from 1990 to 2021. Nevertheless, the most significant dietary contributors are diets rich in sodium and deficient in fruit, with the stroke burden associated with dietary risks remaining especially elevated in Central Europe, East Asia, and Eastern Europe. Lowering sodium consumption and enhancing fruit intake can aid in alleviating the global disease burden.

## 1 Background

Stroke is linked to elevated rates of morbidity, mortality, recurrence, and disability (1). According to the most recent Global Burden of Disease (GBD) Stroke Burden Estimates for 2019, stroke ranks as the second leading cause of death and the third leading cause of disability globally. The worldwide economic repercussions of stroke are staggering, with costs projected to exceed $891 billion, or 1.12% of global GDP. This substantial financial strain imposes a significant burden on both societies and families impacted by stroke (2).

Research indicates that over 90% of strokes can be attributed to modifiable factors such as tobacco use, nutrition, air pollution, and physical inactivity (3, 4). Prospective cohort studies have demonstrated that a nutritious diet, especially the Mediterranean diet, can diminish the incidence of stroke by at least 40% in high-risk individuals (5). Dietary risk is linked to mortality and disability-adjusted life years (DALYs) for a variety of diseases, including cardiovascular diseases, cancer, and type 2 diabetes (6). However, there remains no definitive agreement on the significance of dietary risk as a contributor to death and disability of stroke. Recent studies have highlighted that not only the type but also the quantity and timing of dietary intake play significant roles in cardiovascular health and stroke risk. The field of chrononutrition explores how meal timing and circadian rhythms affect metabolic processes, suggesting that irregular eating patterns may increase cardiovascular risk (7). However, these aspects are often underrepresented in global burden analyses.

Despite numerous studies examining individual dietary components and stroke risk, comprehensive analyses quantifying the global burden of stroke attributable to specific dietary risk factors over time and across diverse populations are limited. Previous research has often focused on specific populations or single dietary factors, which limits the generalizability of the findings and the ability to formulate global strategies.

This gap in the literature underscores the need for a detailed assessment of how dietary risks contribute to stroke burden worldwide. Understanding these relationships is crucial for developing effective prevention strategies and informing public health policies. By analyzing trends over an extended period and across various regions, we can identify priority areas and risk factors that require targeted interventions.

This study aims to fill this gap by specifically assessing the impact of nine dietary risk factors included in the GBD 2021 study on the global burden of ischemic stroke from 1990 to 2021. By providing a comprehensive epidemiological analysis across 204 countries and territories over a 31-year period, we aim to inform stroke prevention strategies and dietary recommendations on a global scale.

## 2 Methods

### 2.1 Sources

All information utilized in this research is derived from the GBD2021 database, which employs a standardized methodology to assess the burden of disease for 88 risk factors across 631 diseases in 204 nations and 811 regions globally. Specifics regarding data sources, estimation techniques, and other pertinent information are detailed in the published series of GBD studies (8). This research relies on a publicly accessible database and does not necessitate ethical approval.

### 2.2 Case definitions

According to the WHO clinical criteria (9), stroke is a swiftly progressing clinical indication of cerebral dysfunction (typically focal) that endures for more than 24 h or culminates in death. Stroke was characterized as an episode of neurological impairment induced by focal cerebral, spinal cord, or retinal infarction. Cerebral hemorrhage was delineated as a stroke accompanied by localized bleeding in the brain that was not attributable to trauma. Subarachnoid hemorrhage was identified as a non-traumatic stroke resulting from bleeding in the subarachnoid space of the brain. In the absence of neuroimaging to facilitate diagnosis, the GBD categorizes causes into four levels, ranging from the most general (level 1, e.g., NCDs) to the most specific (level 4, e.g., cerebral hemorrhage). Stroke is classified as a level 3 cause within the level 2 category of cardiovascular diseases, whereas its subtypes are categorized as level 4 causes. We employed vital registration and verbal autopsy data as inputs to a cause of death ensemble modeling (CODEm) framework to estimate fatalities from total stroke and its subtypes. CODEm is a versatile modeling tool that utilizes geospatial relationships and covariate information to produce estimates of deaths across all locations in a time series (1990–2019). Deaths recorded in vital registration systems classified as unlikely or intermediate causes of death or unspecified stroke were reallocated using statistical methods.

### 2.3 Attributable dietary risk factors

In accordance with the GBD risk factor selection criteria (10–12), we considered several factors. These included the importance of the risk factor for disease burden or policy, the availability of sufficient data to estimate exposure, the robustness of the epidemiological evidence supporting a causal relationship between exposure and disease endpoint, the existence of data quantifying this relationship per unit change in exposure, and the generalizability of these effects across populations. Based on these criteria, we selected nine dietary risk factors: low fruit intake, low vegetable intake, low whole grain intake, high red meat intake, high processed meat intake, high sugar-sweetened beverage intake, low fibre intake, low omega-6 polyunsaturated fatty acid intake, and high sodium intake. These were used to estimate gaps in intake or excess intake of each dietary component. We compared the current intake of each nutrient with the midpoint of its optimal intake range, with high intake defined as the midpoint of an intake above the optimal range, and low intake defined as the midpoint of an intake below the optimal range. The optimal intake ranges and cut-offs in grams per day for each dietary factor were defined according to the GBD 2021 study (9). For example, the optimal intake for fruits is 200–300 g/day, vegetables 290–430 g/day, whole grains 100–150 g/day, red meat 0–23 g/day, processed meat 0–2 g/day, sugar-sweetened beverages 0–2.5 g/day, fibre 24–38 g/day, omega-6 polyunsaturated fatty acids 11–22% of energy intake, and sodium 1–5 g/day. This method allowed us to determine both high and low intakes of dietary factors (6).

### 2.4 Evaluation indicators

Stroke was recognized as the principal health outcome of concern, with dietary risk as a potential contributing factor. The severity of the illness was assessed using several metrics: number of fatalities, DALYs, mortality rate, and DALY rate. These were scrutinized, modeled, and estimated utilizing the Bayesian regression tool DisModMR 2.1. In this study, we focused on total stroke and ischemic stroke, as these subtypes are most strongly associated with dietary risk factors. Data on intracerebral hemorrhage and subarachnoid hemorrhage in relation to dietary risks were less comprehensive in the GBD dataset, which limited our ability to include them in the analysis. Considering the potential variation among age groups, sexes, country regions, and socio-demographic index (SDI) regions, we conducted an age standardization of the data based on the GBD world standard population. In this procedure, particular emphasis was placed on the age-standardized death rate (ASDR) and the DALY rate. By amalgamating their estimated annual percentage change (EAPC) in ASDR and age-standardized DALY rates, we were able to quantify yearly trends in both ASDR and age-standardized DALY rates. This method also enabled us to discern the specific distribution of disease burden among various groups.

### 2.5 Statistical analysis

We meticulously organized and scrutinized the data to illustrate the impact of stroke attributable to dietary risks in our nation over various years, age groups, and sexes. This encompassed the number of deaths, mortality rates, DALYs, and the DALY rate, while also presenting the 95% uncertainty interval (UI) of the distribution. We calculated the age-standardized rates (ASR) and disability-adjusted life years (DALYs) using the standardized methodologies outlined in the GBD 2021 study. The ASR was calculated by applying the age-specific rates to the GBD world standard population, ensuring comparability across regions and over time. DALYs were computed by summing years of life lost (YLL) due to premature mortality and years lived with disability (YLD), following the GBD’s established protocols. We investigated trends in disease burden from 1990 to 2021 by computing the rate of change, defined as (2021 rate–1990 rate)/1990 rate × 100%. Furthermore, trend analysis was conducted utilizing R software (version 4.0.3), and linear regression equations were employed to ascertain the EAPC of standardized mortality and DALY rates, globally, and across various SDI regions for the period 1990–2021. The EAPC was computed using the formula EAPC = 100 × [exp(*β*) – 1], where the value of β signifies the trajectory of change in the age-standardized rate (ASR). When both the EAPC and the 95% confidence interval are positive, the change is upward; when both are negative, the change is downward (13, 14).

## 3 Results

### 3.1 Global burden of stroke disease attributable to dietary risk by age and sex, 2021

**Figure 1:**
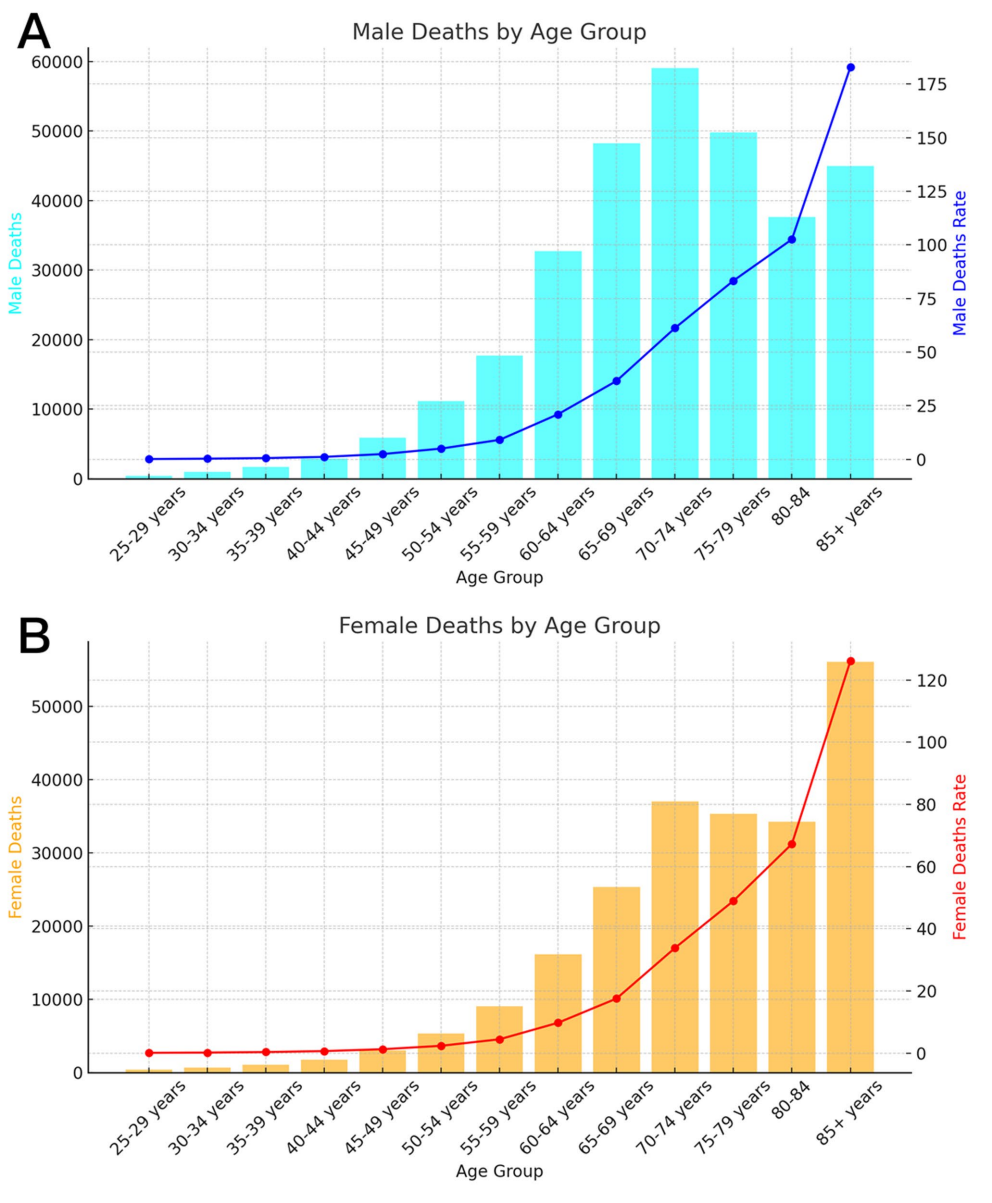
Age-specific numbers and rates of deaths for ischemic stroke attributable to dietary factors by age, by sex, in 2021. **(A)** Male **(B)** Female.

In 2021, global stroke fatalities linked to dietary risk escalated with age, reaching a zenith in the 85+ age category for both sexes at 182.88 per 100,000 deaths for men and 126.10 per 100,000 for female (Figure 1). Nevertheless, the distribution of global stroke fatalities attributable to dietary risk in 2021 exhibited a pronounced sex disparity across age groups: the death toll among men displayed an upward trajectory, followed by a downward trend with advancing age, peaking at 59,037 fatalities in the 70–74 age bracket (Figure 1A). In contrast, the number of deaths among female peaked at 56,073 fatalities in the 85+ age group (Figure 1B). Importantly, global stroke mortality and deaths due to dietary risk were persistently higher in men than in female across all age categories.

**Figure 2:**
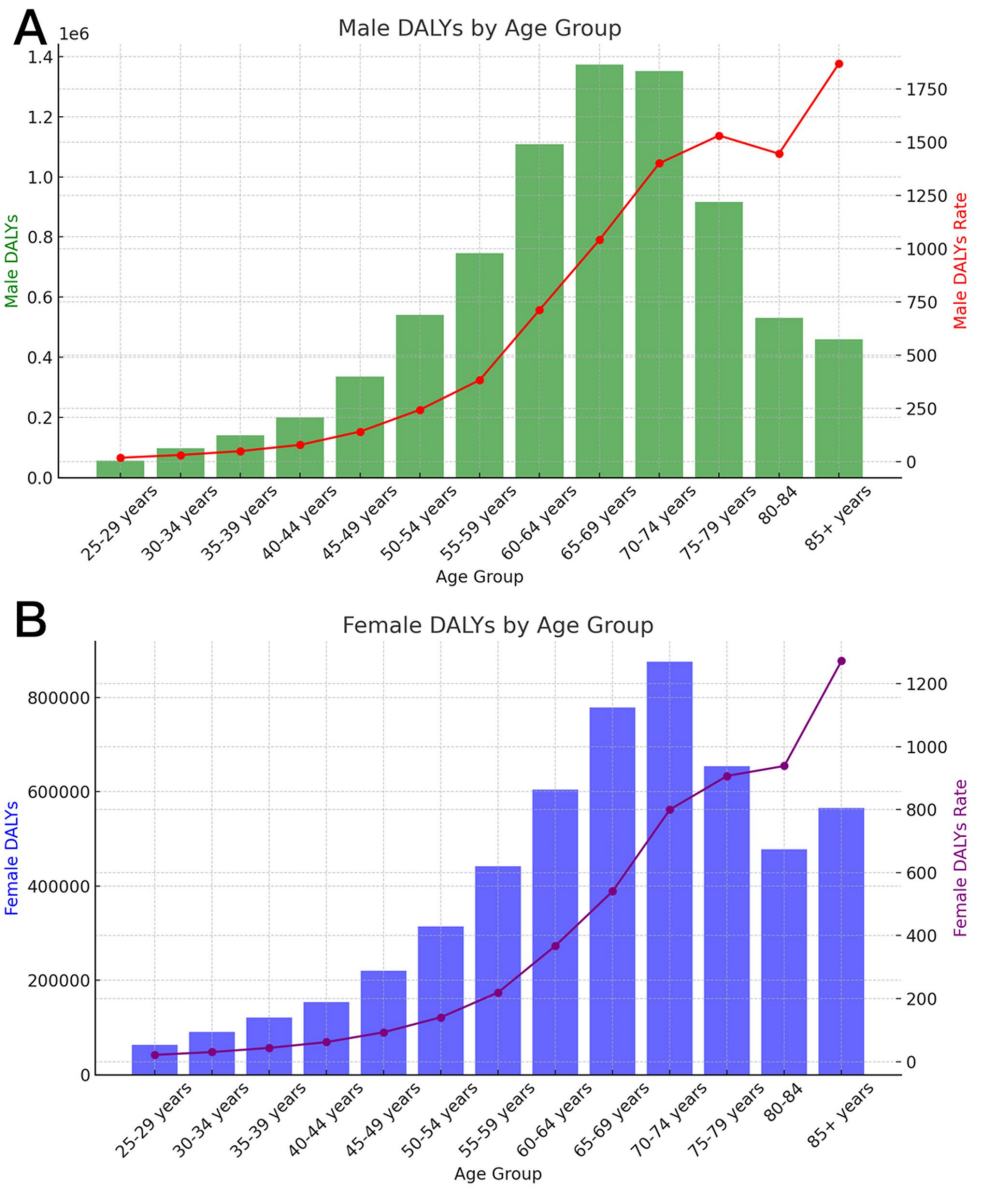
Age-specific numbers and rates of DALY for ischemic stroke attributable to dietary factors by age, by sex, in 2021. **(A)** Male **(B)** Female.

The worldwide DALY attributable to dietary risk for stroke in 2021 exhibited an upward trajectory, subsequently followed by a downward trend as age increased. The peak DALY was recorded in the 65–69-year age bracket (men) and the 70–74-year age bracket (female) (Figure 2). The highest global DALY rate of stroke linked to dietary risk was noted in individuals aged 85 and older, with rates of 1869.22 per 100,000 (men) and 1272.06 per 100,000 (female).

### 3.2 Standardized rate of stroke attributable to dietary risk, by region, globally, 2021

**Figure 3:**
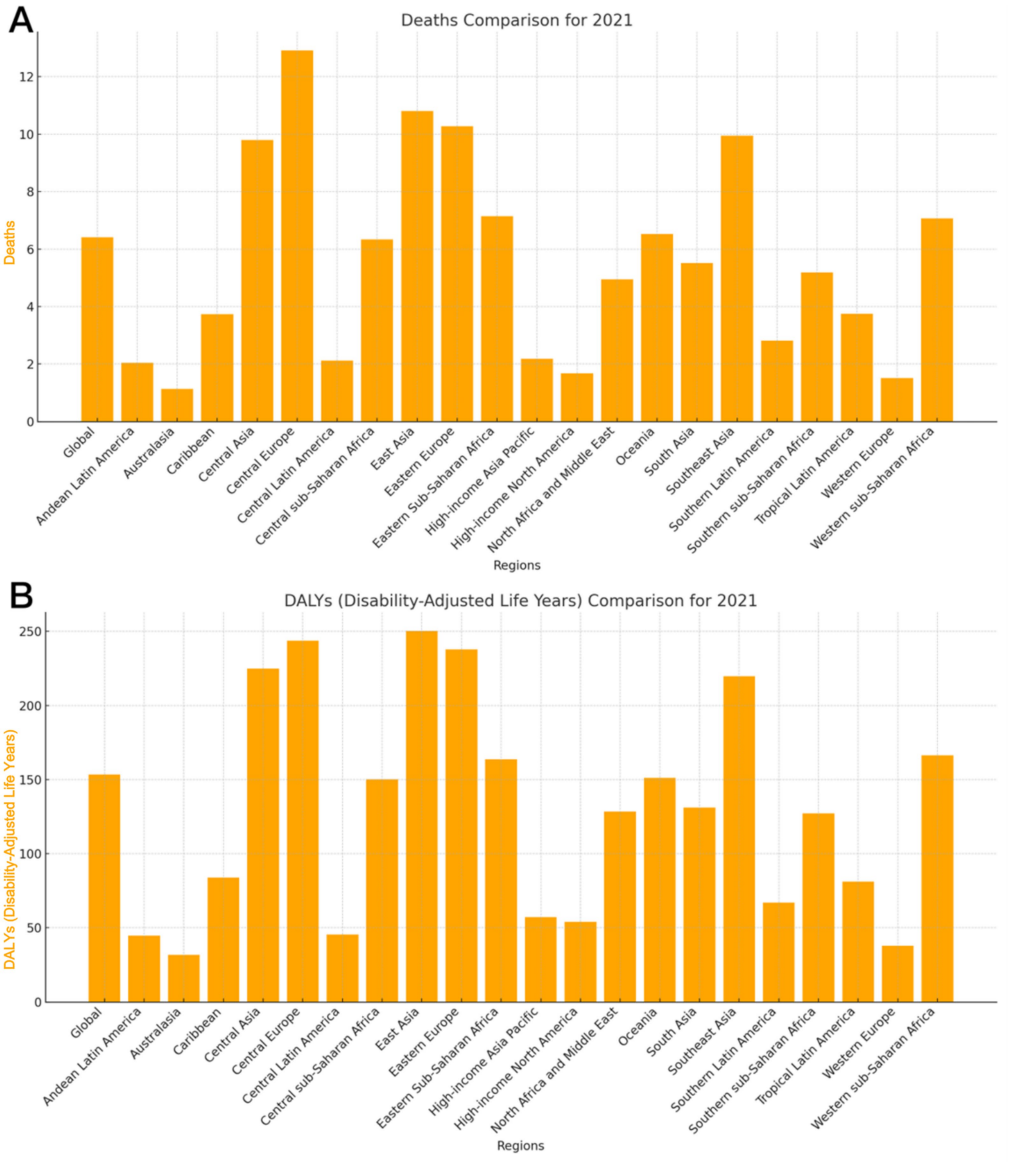
ASRs of global burden for ischemic stroke attributable to dietary factors in 2021, by regions. **(A)** ASDR **(B)** Age-standardized DALY rate. DALY = disability adjusted life-year. ASDR = age standardized Death rate. ASRs = age standardized rates.

The global age-standardized and age-standardized rates of ischemic stroke attributable to dietary risk in 2021 have changed substantially from those in 1990 (Figure 3; Supplementary Table 3). The top five regions with the highest global ASDR for stroke attributable to dietary risk in 2021 were Central Europe (12.92%), East Asia (10.81%), Eastern Europe (10.28%), Southeast Asia (9.95%), and Central Asia (9.80%) (Figure 3A; Supplementary Figure 4). These five regions also ranked as the top five regions with the highest standardized rates in 1990 (Supplementary Figure 3A). The top five regions with the highest global age-standardized DALY rate for stroke attributable to dietary risk in 2021 were East Asia (250.36/100,000), Central Europe (250.36/100,000), Eastern Europe (237.72/ 100,000), Central Asia (224.80/100,000), and Southeast Asia (219.8/100,000) (Figure 3B; Supplementary Figure 5). In contrast, the order of the top five regions was different in 1990: Central Europe (549.84/100,000), Eastern Europe (480.46/100,000), Central Asia (349.06/100,000), East Asia (332.93/100,000), and Southeast Asia (321.51/100,000) (Supplementary Figure 3).

Analysis of the global age-standardized rate of stroke attributable to dietary risk, as classified by the SDI, showed that the ASDR in 2021 was highest in the High-Middle SDI [8.89 (95% UI: 2.08, 16.29)] (Supplementary Figure 2), followed by the Middle SDI [7.68 (95% UI: 1.72, 14.21)], Low-Middle SDI [6.71 (95% UI: 1.29, 12.69)] and Low SDI [6.70 (95% UI: 1.23, 13.21)], with the lowest being the High SDI region [2.45 (95% UI: 0.44, 4.70)]. The trend in DALY rate was as follows: High-Middle SDI [203.25 (95% UI: 57.51, 346.01), 1/100,000], Middle SDI [178.49 (95% UI: 57.12, 303.52), 1/100,000], Low SDI [160.15 (95% UI: 47.56, 284.20), 1/100,000], Low -Middle SDI [156.45 (95% UI: 44.83, 270.35), 1/100,000], and High SDI [66.26 (95% UI: 11.52, 114.69), 1/100,000].

### 3.3 Trends in standardized rates of stroke attributable to dietary risk across global regions, 1990–2021

**Figure 4:**
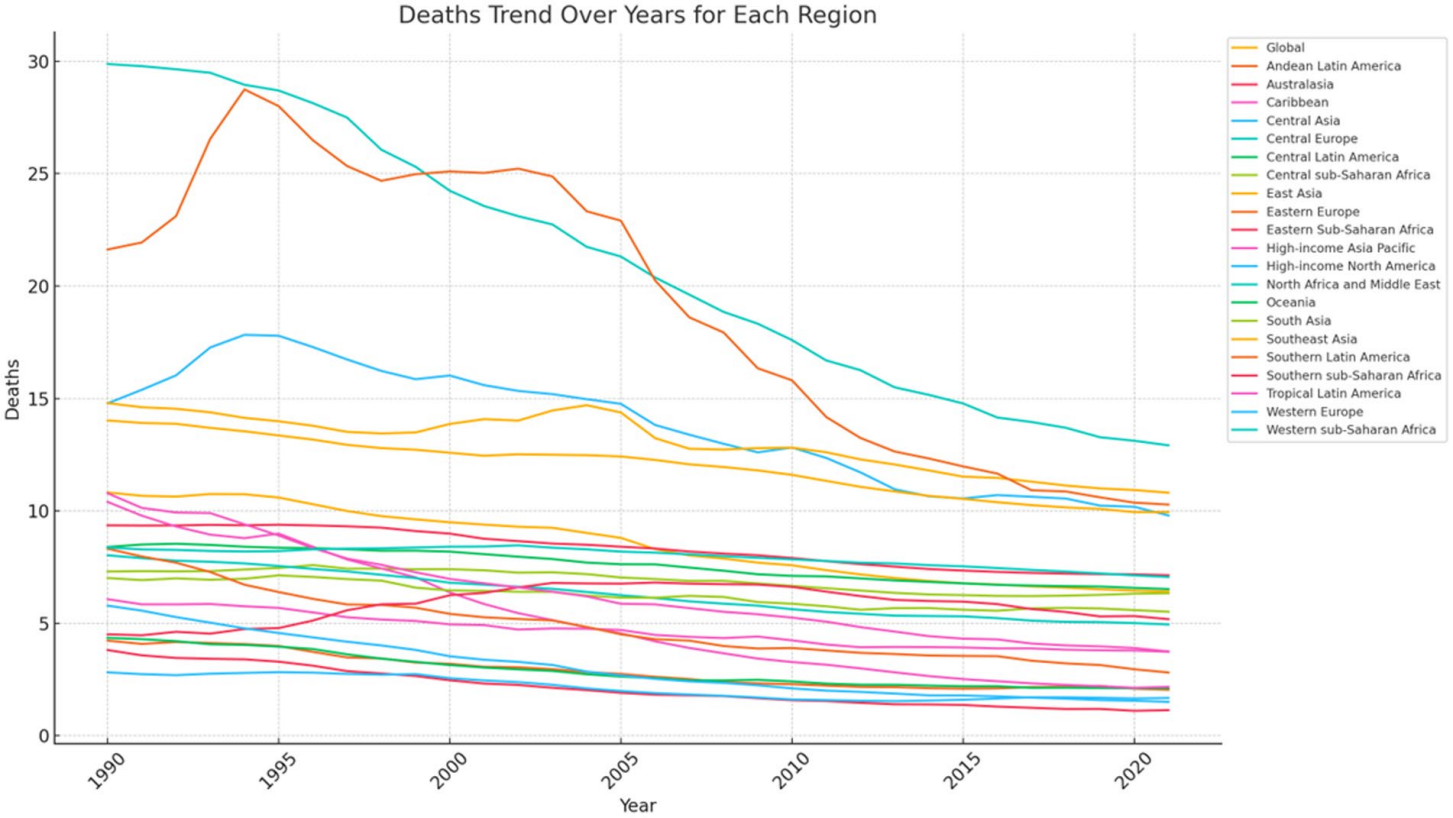
Trends of ASDR of global burden for ischemic stroke attributable to dietary factors from 1990 to 2021, by locations.

**Figure 5:**
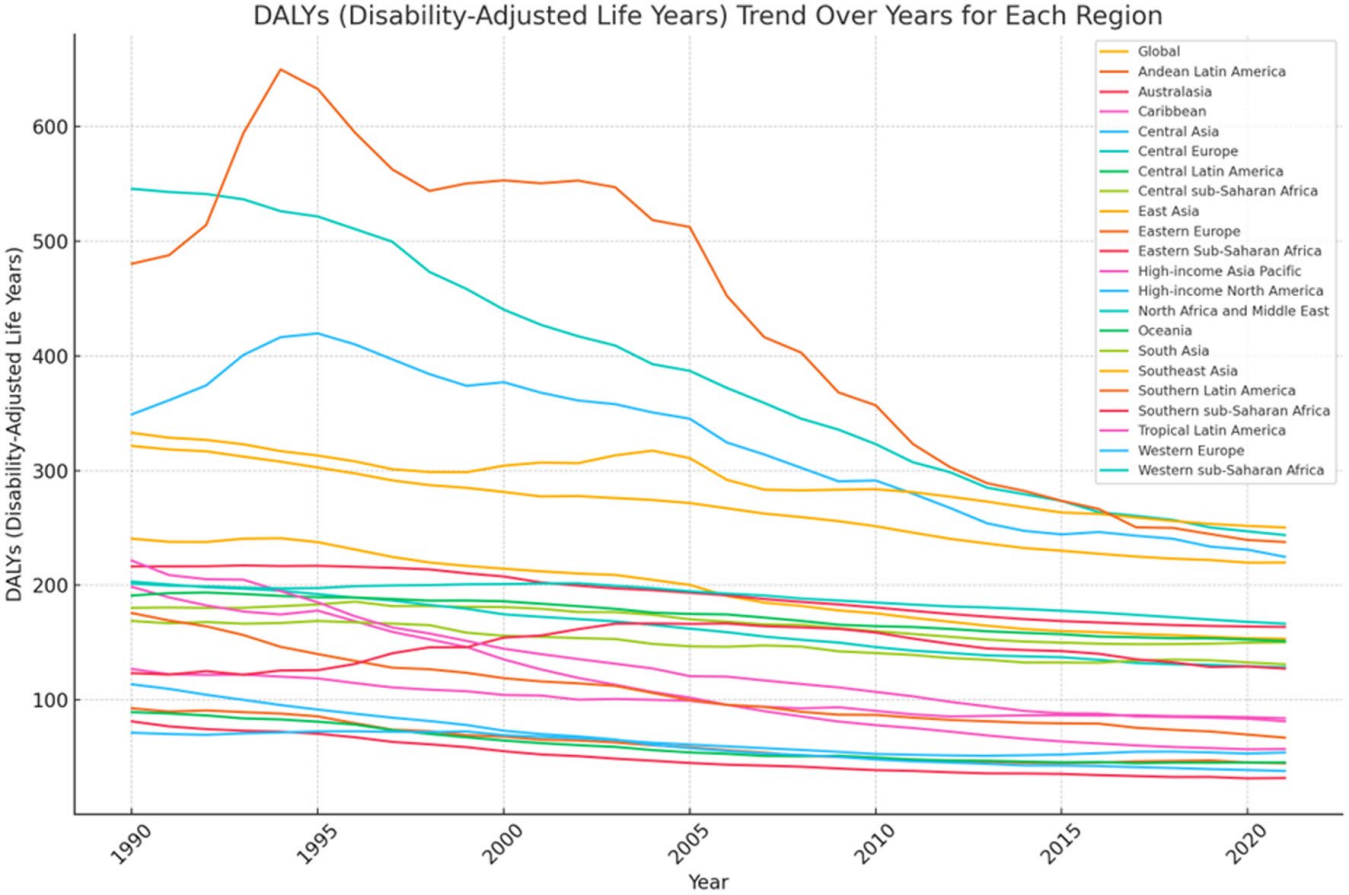
Trends of Age-standardized DALY rate of global burden for ischemic stroke attributable to dietary factors from 1990 to 2021, by locations.

The overall ASDR for stroke (Figure 4; Supplementary Figure 1A) and the overall age-standardized DALY rate attributable to dietary risk (Figure 5; Supplementary Figure 1B) decreased globally from 1990 to 2021. The ASDR and age-standardized DALY rate decreased most significantly in Central Europe, with the ASDR decreasing from 29.88% in 1990 to 12.92% in 2021 and the age-standardized DALY rate decreasing from 545.84 per 100,000 individuals in 1990 to 243.76 per 100,000 individuals in 2021 (Figures 4, 5). Eastern Europe and Central Asia showed an initial increase followed by a decrease in standardized rates between 1990 and 2021. The ASDR for ischemic stroke in Eastern Europe increased from 21.63% to 28.75%, then gradually decreased to 12.92% in 2021. Its age-standardized DALY rate increased from 480.46 per 100,000 individuals to 649.98 per 100,000 individuals, then gradually decreased to 237.72 per 100,000 individuals in 2021. The stroke ASDR in Central Asia increased from 14.78% to 17.79%, then gradually decreased to 9.80% in 2021, with the inflection point occurring in 1995; its age-standardized DALY rate also increased from 349.06 per 100,000 individuals to 419.67 per 100,000 individuals, then gradually decreased to 224.80 per 100,000 individuals in 2021, with the inflection points of both occurring in 1995 and 2021, respectively.

Globally, the ASDR decreased significantly from 1990 to 2021 (Tables 1, 2; Supplementary Figure 1), with an overall EAPC of −1.95 (95% CI: −2.05, −1.84). The decline was more pronounced in female than in men [EAPC: −2.42 (95% CI: −2.56, −2.29)], while the EAPC for ASDR in men showed a decreasing trend [EAPC: −1.60 (95% CI: −1.68, −1.51)].The region with the most significant EAPC for both ASDR and age-standardized DALY rate was High-income North America, with a Death rate EAPC of −5.52 (95% CI: −5.74, −5.29) and a DALY rate EAPC of −4.45 (95% CI:−4.66, −4.25) (Table 2; Supplementary Figure 1). According to the SDI analysis, the absolute value of the EAPC for ASDR was the largest and statistically significant in the High SDI region [EAPC: −3.64 (95% CI: −3.81, −3.47)], followed by the High-middle SDI region [EAPC: −2.42 (95% CI: −2.64, −2.20)] (Table 2; Supplementary Figure 1). Among the 204 countries included in this study, the three countries with the largest EAPCs for both ASDR and age-standardized DALY rate were Estonia (death rate EAPC: −7.36 [95% CI: −8.03, −6.68]; DALY rate EAPC: −6.71 [95% CI: −7.29, −6.13]), Singapore (death rate EAPC: −7.19 [95% CI: −7.64, −6.73]; DALY rate EAPC: −5.90 [95% CI: −6.05, −5.74]), and Luxembourg (death rate EAPC: −6.26 [95% CI: −6.36, −6.17]; DALY rate EAPC: −5.80 [95% CI: −5.94, −5.67]) (Supplementary Table 1).

### 3.4 Standardized rates of ischemic stroke attributable to different dietary risk in 2021

As shown in Table 1, the highest and statistically significant standardized rate of ischemic stroke attributable to a diet high in sodium was observed in 2021 (3.96%; 95% UI) with an ASDR (95% UI) of 3.96% (0.77, 9.23%) and an age-standardized DALY rate of [84.35 (95% UI: 20.33, 187.35), 1/100,000]. The top three regions for ASDR of ischemic stroke attributable to a diet high in sodium in 2021 were Central Europe (9.86), East Asia (8.61), and Southeast Asia (7.41). The top three countries were North Macedonia (33.13%), Bulgaria (23.30%), and Serbia (20.01%), with the highest prevalence of ischemic strokes attributable to a diet high in sodium. The top three regions for age-standardized DALY rate of ischemic stroke attributed to a diet high in sodium were East Asia (187.15), Central Europe (169.10), and Southeast Asia (148.84). The top three countries were North Macedonia (477.26/100,000), Bulgaria (387.58/100,000), and Serbia (317.62/100,000) (Supplementary Tables 2–4).

**Table 1:** Global age-standardized rates and rate change for ischemic stroke attributable to dietary factors, 1990 and 2021.

	DALYs (Disability-adjusted life years)	Deaths	DALYs (Disability-adjusted life years)	Deaths	DALYs (Disability-adjusted life years)	Deaths
	Age-standardized rate per 100, 000 population (95% UI)				Estimated annual percentage change from 1990 to 2019 (95% CI)	
	1990	1990	2021	2021		
Factors	DALY rate	Death rate	DALY rate	Death rate	DALY rate	Death rate
Dietary risks	240.59 (76.68, 407.65)	10.82 (2.63, 19.64)	153.28 (45.74, 259.88)	6.41 (1.45, 11.77)	−1.70 (−1.80, −1.61)	−1.95 (−2.05, −1.84)
Diet low in fruits	41.87 (22.05, 67.60)	1.47 (0.28, 2.76)	23.75 (12.40, 38.17)	0.79 (0.16, 1.52)	−2.17 (−2.30, −2.03)	−2.36 (−2.51, −2.21)
Diet low in vegetables	28.58 (14.26, 45.21)	1.06 (0.10, 2.06)	13.75 (7.05, 21.33)	0.47 (0.06, 0.89)	−2.50 (−2.68, −2.32)	−2.78 (−2.94, −2.63)
Diet low in whole grains	57.19 (−57.78, 164.49)	2.15 (−2.10, 7.35)	35.95 (−36.48, 101.74)	1.21 (−1.15, 3.95)	−1.80 (−1.94, −1.67)	−2.21 (−2.36, −2.05)
Diet high in red meat	−1.53 (−8.66, 14.79)	−0.08 (−0.42, 0.46)	1.30 (−1.64, 13.25)	0.00 (−0.16, 0.35)	–	–
Diet high in processed meat	14.16 (3.19, 25.49)	0.80 (0.18, 1.43)	5.20 (1.21, 9.33)	0.27 (0.06, 0.46)	−4.00 (−4.35, −3.66)	−4.23 (−4.56, −3.91)
Diet high in sugar-sweetened beverages	2.28 (1.07, 3.52)	0.14 (0.07, 0.21)	1.72 (0.83, 2.73)	0.08 (0.04, 0.13)	−1.08 (−1.21, −0.94)	−1.81 (−1.93, −1.68)
Diet low in fiber	21.40 (−0.93, 48.06)	0.75 (−0.12, 1.99)	10.99 (−0.46, 22.36)	0.35 (−0.03, 0.89)	−2.50 (−2.70, −2.30)	−2.87 (−3.10, −2.64)
Diet low in omega-6 polyunsaturated fatty acids	0.32 (0.09, 0.60)	0.02 (0.00, 0.03)	0.21 (0.06, 0.39)	0.01 (0.00, 0.02)	−1.64 (−1.75, −1.54)	−1.89 (−2.00, −1.78)
Diet high in sodium	120.82 (29.89, 263.94)	6.02 (1.28, 13.99)	84.35 (20.33, 187.35)	3.96 (0.77, 9.23)	−1.33 (−1.41, −1.25)	−1.53 (−1.61, −1.44)

**Table 2:** Age-standardized rates and estimated annual percentage change for ischemic stroke attributable to dietary factors, by SDI and regions, 1990 and 2021.

	DALYs (Disability-adjusted life years)	Deaths	DALYs (Disability-adjusted life years)	Deaths	DALYs (Disability-adjusted life years)	Deaths
	Age-standardized rate per 100, 000 population (95% UI)			Estimated annual percentage change from 1990 to 2021 (95% CI)		
	1990	1990	2021	2021		
	DALY rate	Death rate	DALY rate	Death rate	DALY rate	Death rate
Global						
both	240.59 (76.68, 407.65)	10.82 (2.63, 19.64)	153.28 (45.74, 259.88)	6.41 (1.45, 11.77)	−1.70 (−1.80, −1.61)	−1.95 (−2.05, −1.84)
female	203.25 (53.89, 348.76)	9.23 (1.96, 17.39)	116.65 (27.63, 206.27)	4.82 (0.94, 9.33)	−2.11 (−2.23, −1.98)	−2.42 (−2.56, −2.29)
male	285.53 (97.12, 476.34)	12.88 (3.47, 23.13)	195.27 (62.34, 324.05)	8.36 (2.01, 15.00)	−1.42 (−1.50, −1.34)	−1.60 (−1.68, −1.51)
SDI						
High-middle SDI	353.54 (80.72, 609.16)	16.42 (3.71, 30.17)	203.25 (57.51, 346.01)	8.89 (2.08, 16.29)	−2.23 (−2.44, −2.03)	−2.42 (−2.64, −2.20)
High SDI	142.76 (33.39, 251.80)	6.80 (1.38, 13.06)	66.26 (11.52, 114.69)	2.45 (0.44, 4.70)	−2.74 (−2.89, −2.60)	−3.64 (−3.81, −3.47)
Low-middle SDI	205.73 (74.91, 350.25)	8.69 (2.11, 16.10)	156.45 (44.83, 270.35)	6.71 (1.29, 12.69)	−0.97 (−1.02, −0.92)	−0.91 (−0.97, −0.86)
Low SDI	200.92 (66.28, 356.51)	8.36 (1.98, 16.53)	160.15 (47.56, 284.20)	6.70 (1.23, 13.21)	−0.87 (−0.94, −0.79)	−0.81 (−0.88, −0.74)
Middle SDI	257.13 (96.31, 419.63)	11.29 (3.26, 20.03)	178.49 (57.12, 303.52)	7.68 (1.72, 14.21)	−1.23 (−1.29, −1.18)	−1.31 (−1.37, −1.25)
Regions						
Andean Latin America	92.64 (20.67, 168.35)	4.23 (0.53, 8.47)	44.72 (8.91, 85.40)	2.04 (0.23, 4.25)	−2.63 (−2.88, −2.37)	−2.62 (−2.86, −2.37)
Australasia	81.18 (−0.90, 162.74)	3.81 (−0.13, 8.48)	31.84 (−2.09, 62.29)	1.14 (−0.02, 2.54)	−3.17 (−3.35, −2.98)	−4.08 (−4.23, −3.93)
Caribbean	126.95 (18.49, 232.84)	6.07 (0.64, 12.28)	83.96 (11.94, 162.61)	3.73 (0.43, 8.01)	−1.39 (−1.51, −1.27)	−1.64 (−1.74, −1.54)
Central Asia	349.06 (10.28, 612.65)	14.78 (1.94, 26.75)	224.80 (−33.99, 445.02)	9.80 (−0.42, 20.07)	−2.04 (−2.32, −1.75)	−1.92 (−2.19, −1.65)
Central Europe	545.84 (187.54, 875.25)	29.88 (9.72, 48.85)	243.76 (73.34, 405.06)	12.92 (3.76, 22.03)	−2.92 (−3.03, −2.81)	−3.03 (−3.15, −2.91)
Central Latin America	89.29 (25.50, 161.19)	4.35 (0.95, 8.60)	45.40 (11.59, 82.89)	2.11 (0.42, 4.23)	−2.46 (−2.71, −2.21)	−2.57 (−2.80, −2.35)
Central Sub-Saharan Africa	180.25 (65.47, 315.45)	7.30 (1.22, 14.46)	150.28 (45.50, 286.82)	6.34 (0.78, 13.56)	−0.83 (−0.93, −0.73)	−0.70 (−0.81, −0.60)
East Asia	332.93 (130.72, 553.16)	14.80 (4.88, 26.33)	250.36 (80.08, 423.79)	10.81 (2.72, 19.68)	−0.87 (−0.98, −0.77)	−0.98 (−1.14, −0.82)
Eastern Europe	480.46 (7.18, 899.37)	21.63 (0.98, 43.35)	237.72 (5.16, 452.39)	10.28 (0.62, 21.52)	−3.30 (−3.84, −2.75)	−3.43 (−4.00, −2.86)
Eastern Sub-Saharan Africa	216.42 (80.19, 370.44)	9.36 (2.60, 17.50)	163.61 (54.78, 277.51)	7.14 (1.59, 13.46)	−1.10 (−1.18, −1.03)	−1.06 (−1.13, −0.99)
High-income Asia Pacific	198.61 (64.51, 340.71)	10.40 (2.83, 18.65)	57.17 (15.51, 100.53)	2.18 (0.48, 4.21)	−4.45 (−4.66, −4.25)	−5.52 (−5.74, −5.29)
High-income North America	71.31 (2.30, 134.69)	2.82 (0.34, 5.95)	54.07 (5.17, 97.85)	1.68 (0.21, 3.48)	−1.30 (−1.52, −1.08)	−2.30 (−2.67, −1.93)
North Africa and Middle East	203.16 (−72.04, 444.15)	8.03 (−2.49, 19.47)	128.30 (−51.59, 290.27)	4.95 (−1.92, 12.50)	−1.61 (−1.66, −1.56)	−1.68 (−1.75, −1.62)
Oceania	191.03 (52.72, 339.92)	8.40 (1.75, 15.73)	151.30 (35.91, 272.21)	6.52 (1.06, 12.81)	−0.89 (−0.95, −0.84)	−0.98 (−1.05, −0.92)
South Asia	168.83 (66.49, 295.36)	7.01 (1.54, 13.37)	131.02 (45.04, 230.46)	5.51 (1.06, 10.46)	−0.95 (−1.03, −0.87)	−0.91 (−1.01, −0.82)
Southeast Asia	321.51 (142.22, 496.79)	14.02 (4.82, 23.62)	219.81 (74.14, 396.53)	9.95 (2.38, 19.45)	−1.29 (−1.34, −1.25)	−1.14 (−1.21, −1.06)
Southern Latin America	175.75 (−4.32, 332.26)	8.32 (0.19, 16.58)	67.05 (−0.83, 128.20)	2.81 (0.12, 5.74)	−2.95 (−3.11, −2.79)	−3.20 (−3.36, −3.03)
Southern Sub-Saharan Africa	123.33 (48.72, 206.76)	4.51 (0.67, 8.80)	127.26 (46.81, 222.41)	5.19 (0.63, 10.44)	0.21 (−0.23, 0.65)	0.59 (0.06, 1.12)
Tropical Latin America	221.47 (53.90, 400.67)	10.79 (1.73, 21.17)	81.25 (11.91, 151.11)	3.75 (0.52, 7.62)	−3.30 (−3.46, −3.13)	−3.34 (−3.47, −3.21)
Western Europe	113.58 (−0.13, 229.30)	5.79 (0.07, 12.81)	37.97 (−0.51, 74.33)	1.51 (−0.05, 3.30)	−3.63 (−3.84, −3.43)	−4.47 (−4.66, −4.29)
Western Sub-Saharan Africa	201.46 (36.01, 394.83)	8.37 (0.71, 18.27)	166.46 (34.03, 313.70)	7.06 (0.74, 14.78)	−0.61 (−0.71, −0.52)	−0.53 (−0.63, −0.42)

The second highest dietary risk was a low fruit diet, with the 2021 ASDR (95% UI) compared to 1990 being [0.79% (95% UI: 0.16, 1.52%) vs. 1.47% (95% UI: 0.28, 2.76%)], and the age-standardized DALY rate being [23.75 (95% UI: 12.40, 38.17) vs. 41.87 (95% UI: 22.05, 67.60), 1/100,000] (Table 1). The top three regions in terms of ASDR for ischemic stroke attributable to a low fruit diet in 2021 were South Asia (2.10%), Southern sub-Saharan Africa (1.84%), and Western sub-Saharan Africa (1.37%); the top three countries were Gambia (6.77%), Togo (5.39%), and Chad (4.51%). The top three regions with an age-standardized DALY rate were Southern sub-Saharan Africa (50.87/100,000), Eastern Europe (36.30/100,000), and Eastern Sub-Saharan Africa (33.61/100,000); the top three countries with an age-standardized DALY rate were Gambia (183.21/100,000), Togo (149.67/100,000), and Chad (123.72/100,000) (Supplementary Tables 2–4).

## 4 Discussion

Our study provides a detailed global and regional analysis of the stroke burden attributable to specific dietary risk factors over a 31-year period using data from GBD 2021. By identifying the most impactful dietary risks and the populations most affected, our findings contribute valuable insights that can inform targeted interventions and public health strategies. The findings revealed substantial disparities in age, sex, and region concerning the global burden of stroke disease attributed to dietary risk. The burden escalated with age, particularly among individuals aged 85 years or older. Furthermore, the burden was greater in men than in female across all age categories. The regions with the most significant burden of stroke disease due to dietary risk included Central Europe, East Asia, Eastern Europe, and Southeast Asia. A high sodium intake has been identified as the most critical dietary risk factor for ischemic stroke.

Our analysis reveals substantial disparities in stroke burden attributable to dietary risks across different age groups, sexes, and regions. These insights provide valuable information that can guide public health policies and strategies. The higher burden observed in older adults and males suggests that interventions should be tailored to these demographics to maximize effectiveness. Furthermore, the identification of specific regions with the highest burden highlights where resources and efforts can be most effectively directed.

The occurrence of stroke is frequently associated with age. A cross-sectional investigation, which encompassed 480,687 Chinese participants, revealed that the prevalence, morbidity, and mortality rates of stroke rise in conjunction with advancing age (15). However, other research indicates that dietary practices significantly affect the onset and progression of age-related vascular ailments (16). Consistent with our findings, global stroke mortality linked to dietary risk in 2021 escalates with age and peaks for both sexes at ages 85 and above. The metabolic efficiency of the body diminishes with age. It has been demonstrated that genes involved in lipid and carbohydrate metabolism exhibit considerable genetic variability, which declines with age, particularly in adipose tissue where PPARγis dysregulated (17). This may explain the age-related escalation in stroke disease burden attributed to dietary risk.

Disparities between sexes are frequently linked to physical and physiological traits such as chromosomes, gene expression, hormone levels and functions, along with reproductive and sexual anatomy. Moreover, the bioavailability of food and nutrients, metabolism, distribution, and excretion of nutrients, as well as nutritional needs across different life stages, demonstrate sex-specific variations (18). Our findings revealed that the age-specific composition of stroke fatalities attributable to dietary risk varied significantly between sexes. The number of male fatalities escalated with age, peaking in the 70–74 years age group before declining. Conversely, the number of female fatalities peaked in the 85+ years age group. The highest Disability-Adjusted Life Years (DALY) were recorded in the 65–69 years age group for men and in the 70–74 years age group for female, indicating that the burden of stroke disease attributable to dietary risk escalates earlier in men than in female. We also observed that the reduction in the burden of stroke disease attributable to dietary risk from 1990 to 2021 was more pronounced in female than in men. Biological sex differences significantly influence the burden of stroke disease. The production of estrogen in female offers physiological protection against cardiovascular disease, a benefit absents in men. As most female undergo menopause around the ages of 45–50 years, fluctuations in estrogen levels may significantly contribute to the increased risk of stroke (19). This results in a non-overlapping age distribution of stroke burden between sexes. Men consistently exhibit a higher stroke burden than female across all ages. This disparity may be attributed to differences in dietary habits between men and female: a prospective cohort study involving 55,061 participants from the Danish Diet, Cancer, and Health cohort indicated that female demonstrated superior adherence to the Prudent dietary pattern, which is advantageous for stroke prevention, compared to men (20). Furthermore, men may encounter greater exposure to cardiovascular disease risk factors, including excessive alcohol consumption, smoking, diets high in salt, and those rich in cholesterol (21).

By quantifying the impact of specific dietary risk factors on stroke burden over an extended period and across diverse populations, our study adds to the medical knowledge base. This comprehensive approach allows for the evaluation of temporal trends and the effectiveness of past interventions, providing a foundation for future research and policy development. Our findings highlight the critical need for targeted dietary recommendations and interventions to reduce the global burden of stroke.

Factors contributing to these regional disparities may include differences in dietary patterns, socioeconomic status, access to healthcare, public health policies, and genetic predispositions (20). For instance, diets high in sodium are more prevalent in East Asian countries due to traditional culinary practices, which may contribute to the higher burden observed in this region.

Our findings indicate that the global ASDR and age-standardized DALY rate of stroke linked to dietary risk have been persistently declining from 1990 to 2021 across all regions. These patterns signify considerable worldwide advancements in alleviating the burden of stroke associated with dietary risk, likely strongly tied to enhanced dietary habits, evolved health perceptions, and the enactment of policies on a global scale (22). The outcomes are likely closely connected to shifts in dietary patterns and health perceptions internationally, as well as local regulations. Prior research has demonstrated that the disease-specific burden of each dietary risk among adults aged 25 and older varied significantly by region. The findings of the current study align with those of previous investigations. In line with this study’s results, the EAPCs for standardized and age-standardized rates of stroke attributable to dietary risk were globally diverse in 2021, with the top five regions exhibiting the highest standardized mortality rate and age-standardized DALY rate for stroke being Central Europe, East Asia, Eastern Europe, Southeast Asia, and Central Europe. The same regions were identified in 1990 in Europe, Southeast Asia, and Central Asia. Based on the SDI classification analysis, the High-Middle SDI regions recorded the highest global standardized stroke rate linked to dietary risk and age-standardized DALY rate, while the High SDI regions reported the lowest. However, it is important to note that the High SDI regions exhibited the highest ASDR EAPC and age-standardized DALY rate EAPC in absolute terms, indicating that the reduction in stroke disease burden related to dietary risk was most pronounced in the High SDI region.

The ASDR and age-standardized DALY rate for ischemic stroke associated with a high-sodium diet diminished in comparison to those in 1990. The second most significant dietary risk was inadequate fruit consumption. Comparative risk evaluations of global, regional, and national data from 1990 to 2021 concerning 79 behavioral, environmental, occupational, and metabolic risks or risk clusters indicated that environmental risk and malnutrition consistently declined alongside the SDI. Low physical activity, elevated BMI, and high fasting glucose also fell as SDI rose (23). Early economic progress in Central Europe, East Asia, and Southeast Asia resulted in a greater consumption of high salt and animal fats compared to Low SDI nations. This contributed to an increased disease burden linked to food risks for stroke. However, as further research explored the connection between food risks and disease, and as media platforms began to advocate for healthy dietary practices, a notable transformation in dietary habits occurred. This transformation led to a swift reduction in the disease burden associated with stroke in High SDI countries.

The study examined 204 nations (24), and Estonia demonstrated the most pronounced decline in ASDR and age-standardized DALY rate, which may be ascribed to a structural shift in alcohol consumption patterns (25). Diet considerably influences the risk of stroke, alongside other contributing factors such as diabetes, hypertension, and dyslipidemia (26). Despite the drawbacks of dietary studies, including recall bias and measurement inaccuracies, certain elements of diet and nutrition are recognized risk factors for stroke (27). For instance, sodium consumption is linked to an elevated risk of hypertension and stroke. Research suggests that a diet reminiscent of the Mediterranean, or one rich in fruits and vegetables, may help alleviate the risk of stroke (28, 29). However, the remaining studied dietary risk factors, such as high processed meat intake and high red meat intake, appeared less prominent in contributing to the stroke burden in our study. This could be due to lower consumption levels of processed and red meats in certain regions, variations in the strength of associations between these dietary factors and stroke risk, and potential methodological considerations in the GBD estimates. Therefore, a diet defined by low sodium and high consumption of fruits and vegetables may play a crucial role in reducing the dietary risk associated with the disease burden of stroke.

This research represents the inaugural effort to furnish a comprehensive overview of spatial and temporal trends in the stroke disease burden attributable to dietary risks across 204 nations and 811 regions from 1990 to 2021, along with age-sex disparities in diet-related stroke. These insights can inform the development and execution of targeted dietary interventions aimed at diminishing stroke morbidity and mortality. While our study focuses on specific dietary risk factors quantified in the GBD 2021 study, it is important to acknowledge the growing evidence on the impact of dietary quantity, quality, and meal timing on stroke risk. Chronobiology research indicates that misalignment between meal times and the body’s circadian rhythms can adversely affect cardiovascular health (30). Future studies integrating these factors could provide a more comprehensive understanding of diet-related stroke risk.

Our study has several limitations. First, the GBD 2021 relies on mixed sources of dietary data, which are not available for all countries, increasing the statistical uncertainty of our estimates. Nevertheless, this study is not without its limitations. The impact of dietary risk factors on stroke outcomes was predominantly assessed through meta-analyses of prospective observational studies. Although the majority of dietary relative risks were adjusted for significant confounders (e.g., age, sex, smoking, physical activity), the possibility of residual confounding cannot be dismissed. Furthermore, to mitigate the influence of energy intake as a potential confounder and minimize measurement error in dietary evaluation, many cohorts adjust their statistical models for total energy consumption. Consequently, the data do not accurately represent absolute nutrient intake levels (31). Additionally, as the consumption of healthy dietary components is generally positively associated with one another and negatively correlated with unhealthy dietary components, the true effect size of individual dietary factors may be exaggerated.

Second, we focused on total stroke and ischemic stroke due to the availability and strength of data linking these subtypes to dietary risk factors. Our study focused on total stroke and ischemic stroke due to the stronger and more consistent associations between these subtypes and dietary risk factors in existing literature. Additionally, the GBD dataset provided more comprehensive data for these subtypes compared to intracerebral hemorrhage and subarachnoid hemorrhage. Data on intracerebral hemorrhage or subarachnoid hemorrhage were less comprehensive, which limits the generalizability of our findings to all stroke types. Future studies should aim to include these subtypes as more data become available.

Additionally, our analysis does not account for factors such as dietary quantity, meal timing, and chronobiology due to data limitations in the GBD study. These factors may influence stroke risk and should be considered in future research.

The findings of our study have significant policy implications. Governments and health organizations can use this information to prioritize resources and design interventions that address the most impactful dietary risk factors in regions with the highest burden. Policies may include: implementing regulations to reduce sodium content in processed foods, promoting public education campaigns to raise awareness of the risks associated with high sodium intake and low fruit consumption, providing incentives for the production and consumption of fruits and vegetables, developing guidelines for healthy eating that are culturally appropriate and accessible and by tailoring dietary recommendations based on our findings, policymakers can develop more effective strategies to reduce stroke morbidity and mortality.

Our study highlights the need for ongoing research to further understand the barriers to dietary changes in high-risk populations. Future studies should explore the socioeconomic, cultural, and environmental factors influencing dietary habits. Additionally, integrating considerations of dietary quantity, quality, and timing could provide a more comprehensive understanding of diet-related stroke risk.

Public health strategies should also consider the role of education in changing dietary behaviors. Interventions that involve community engagement and address misconceptions about diet and health may enhance the effectiveness of dietary recommendations. The worldwide burden of stroke linked to dietary risks diminished from 1990 to 2021 across all regions, with a more significant decline noted among female compared to men. The most substantial reductions were observed in affluent North America and Estonia, respectively. Nevertheless, the burden of stroke associated with dietary risks continued to be elevated in Central Europe, East Asia, and Eastern Europe. Lowering dietary sodium intake and enhancing fruit consumption could assist in alleviating this burden. Age-, sex-, and region-specific public health initiatives and dietary recommendations should be formulated and enacted to further decrease stroke morbidity and mortality.

## Data Availability

The original contributions presented in the study are included in the article/Supplementary material, further inquiries can be directed to the corresponding author.
