# Supplementary material for: Global burden of stroke attributable to dietary risk factors in the GBD 2021 study
Source: Front Nutr. 2024 Dec 30;11:1494574. doi: 10.3389/fnut.2024.1494574 (PMC11727091; doi:10.3389/fnut.2024.1494574)

stable1.Age-standardized rates and estimated annual percentage change for ischemic stroke attributable to dietary factors, by countries, 1990 and 2021

|  | DALYs (Disability-Adjusted Life Years) | Deaths | DALYs (Disability-Adjusted Life Years) | Deaths | DALYs (Disability-Adjusted Life Years) | Deaths |
| --- | --- | --- | --- | --- | --- | --- |
|  | Age-standardized rate per 100 000 population (95% UI) | | Age-standardized rate per 100 000 population (95% UI) | | Estimated annual percentage change from 1990 to 2021 (95% CI) | |
|  | 1990 | 1990 | 2021 | 2021 |  |  |
|  | DALY rate | Death rate | DALY rate | Death rate | DALY rate | Death rate |
| Afghanistan | 451.53(67.27,878.65) | 17.25(1.34,37.95) | 410.36(48.35,789.17) | 15.54(0.63,33.66) | -0.55(-0.84,-0.25) | -0.57(-0.86,-0.28) |
| Albania | 249.91(89.38,410.69) | 13.32(4.67,22.70) | 168.51(46.59,283.72) | 9.82(2.92,17.53) | -0.95(-1.24,-0.66) | -0.58(-0.90,-0.25) |
| Algeria | 185.74(-40.18,416.37) | 7.85(-1.89,20.57) | 112.16(-44.96,264.89) | 4.73(-1.74,13.20) | -1.87(-1.96,-1.78) | -1.73(-1.80,-1.67) |
| American Samoa | 121.86(17.95,228.66) | 5.04(0.45,10.62) | 104.63(16.63,190.95) | 4.32(0.50,8.90) | -0.79(-0.95,-0.64) | -0.87(-1.07,-0.67) |
| Andorra | 56.12(-3.33,118.02) | 2.59(-0.02,6.11) | 28.06(-2.74,57.01) | 1.13(-0.02,2.66) | -2.12(-2.33,-1.91) | -2.39(-2.66,-2.12) |
| Angola | 220.17(62.79,397.43) | 8.84(1.13,18.11) | 125.49(15.94,255.97) | 5.76(0.31,13.03) | -2.60(-2.90,-2.29) | -2.13(-2.42,-1.84) |
| Antigua and Barbuda | 124.34(-6.60,249.31) | 6.39(-0.24,14.06) | 71.61(-3.02,149.85) | 3.69(-0.30,8.60) | -2.05(-2.26,-1.83) | -2.06(-2.31,-1.82) |
| Argentina | 171.28(-8.06,327.81) | 7.99(0.06,16.07) | 65.93(-2.56,127.05) | 2.63(0.07,5.39) | -2.89(-3.12,-2.67) | -3.23(-3.49,-2.98) |
| Armenia | 247.73(-2.81,464.62) | 11.69(0.83,22.61) | 138.36(-26.08,286.29) | 6.25(-0.28,13.60) | -2.92(-3.30,-2.53) | -3.16(-3.59,-2.73) |
| Australia | 79.36(-1.58,160.18) | 3.72(-0.21,8.33) | 30.58(-2.44,59.65) | 1.05(-0.01,2.37) | -3.23(-3.42,-3.03) | -4.26(-4.42,-4.10) |
| Austria | 141.88(18.19,272.76) | 7.56(0.61,15.69) | 42.61(4.35,81.60) | 1.53(0.19,3.20) | -4.02(-4.41,-3.63) | -5.49(-5.91,-5.07) |
| Azerbaijan | 178.88(-5.60,344.61) | 7.54(0.65,15.15) | 112.12(-20.78,223.48) | 4.93(-0.34,10.37) | -1.64(-1.89,-1.40) | -1.21(-1.48,-0.94) |
| Bahamas | 82.92(-7.60,169.75) | 3.98(-0.20,8.68) | 57.44(-7.15,119.97) | 2.65(-0.20,6.13) | -1.26(-1.37,-1.16) | -1.44(-1.60,-1.29) |
| Bahrain | 128.27(-52.57,308.51) | 5.74(-2.60,15.61) | 67.77(-25.19,168.77) | 3.11(-1.11,8.51) | -2.67(-2.99,-2.34) | -2.58(-2.99,-2.17) |
| Bangladesh | 289.54(128.09,505.06) | 12.86(2.95,24.48) | 191.23(54.42,350.54) | 9.73(0.96,19.91) | -1.34(-1.53,-1.15) | -0.94(-1.25,-0.63) |
| Barbados | 121.72(18.26,237.06) | 6.05(0.00,13.64) | 68.10(4.55,145.95) | 3.39(-0.19,8.30) | -2.03(-2.31,-1.76) | -2.08(-2.37,-1.79) |
| Belarus | 325.86(-84.85,650.93) | 12.48(-2.46,26.73) | 196.49(-54.80,409.86) | 7.52(-1.65,17.36) | -2.50(-2.95,-2.05) | -2.52(-2.97,-2.06) |
| Belgium | 117.77(15.10,220.62) | 6.72(0.66,13.75) | 41.46(1.76,80.30) | 1.78(0.16,3.76) | -3.24(-3.40,-3.08) | -4.11(-4.25,-3.97) |
| Belize | 64.96(7.40,125.99) | 3.18(0.26,6.75) | 49.67(4.12,100.63) | 2.42(0.10,5.42) | -1.13(-1.59,-0.68) | -1.26(-1.81,-0.71) |
| Benin | 227.60(55.85,432.24) | 9.95(1.12,20.83) | 167.47(35.82,325.79) | 7.65(0.79,16.86) | -1.12(-1.27,-0.97) | -0.92(-1.05,-0.79) |
| Bermuda | 90.05(-6.07,191.45) | 4.69(-0.07,10.59) | 40.40(-2.20,84.65) | 1.87(-0.05,4.39) | -2.55(-2.79,-2.32) | -3.07(-3.28,-2.87) |
| Bhutan | 136.80(46.96,257.36) | 5.90(1.20,12.50) | 90.66(20.99,176.84) | 4.27(0.63,9.35) | -1.29(-1.39,-1.19) | -0.94(-1.05,-0.84) |
| Bolivia (Plurinational State of) | 135.55(22.53,276.13) | 6.51(0.82,14.09) | 70.93(14.08,140.42) | 3.52(0.39,7.71) | -2.10(-2.29,-1.92) | -1.96(-2.13,-1.80) |
| Bosnia and Herzegovina | 566.10(225.96,918.95) | 28.44(9.52,47.47) | 323.77(101.04,558.81) | 17.33(5.13,30.43) | -2.20(-2.41,-1.99) | -2.00(-2.24,-1.76) |
| Botswana | 238.54(73.71,417.17) | 10.06(0.85,20.58) | 142.13(42.47,254.31) | 5.65(0.40,11.64) | -1.58(-1.77,-1.39) | -1.71(-1.94,-1.47) |
| Brazil | 223.14(54.55,404.31) | 10.87(1.76,21.35) | 81.03(11.94,150.99) | 3.73(0.50,7.61) | -3.33(-3.49,-3.17) | -3.38(-3.51,-3.26) |
| Brunei Darussalam | 271.21(86.98,465.63) | 12.31(2.87,23.11) | 121.27(34.95,214.60) | 5.53(1.12,10.70) | -2.58(-2.80,-2.37) | -2.33(-2.62,-2.03) |
| Bulgaria | 740.71(267.10,1181.52) | 41.89(14.19,68.00) | 571.63(158.70,958.20) | 30.78(8.97,53.11) | -0.69(-0.86,-0.53) | -0.79(-0.95,-0.62) |
| Burkina Faso | 196.04(66.72,364.57) | 7.74(0.98,16.07) | 188.78(71.79,332.61) | 7.66(1.47,15.54) | 0.13(0.05,0.22) | 0.29(0.16,0.42) |
| Burundi | 345.31(99.03,633.13) | 15.26(3.32,30.04) | 180.06(40.92,353.64) | 8.04(1.24,17.06) | -2.73(-3.00,-2.45) | -2.72(-3.01,-2.44) |
| Cabo Verde | 145.12(49.18,251.59) | 5.41(0.87,10.77) | 117.74(15.67,251.18) | 5.18(0.25,12.21) | -1.06(-1.49,-0.64) | -0.57(-1.06,-0.07) |
| Cambodia | 336.82(151.83,553.49) | 15.48(5.13,27.75) | 245.71(93.77,416.56) | 12.03(2.88,22.43) | -1.23(-1.34,-1.13) | -1.00(-1.11,-0.89) |
| Cameroon | 128.74(21.26,271.11) | 5.52(0.42,12.70) | 125.17(15.10,278.97) | 5.59(0.43,13.47) | -0.22(-0.66,0.22) | -0.04(-0.48,0.41) |
| Canada | 66.74(2.91,135.67) | 2.84(0.07,6.66) | 32.89(1.82,65.86) | 0.94(0.03,2.23) | -2.58(-2.77,-2.38) | -4.01(-4.22,-3.79) |
| Central African Republic | 239.26(84.07,452.54) | 10.09(1.75,21.19) | 219.19(78.00,407.76) | 9.51(1.25,19.55) | -0.40(-0.49,-0.31) | -0.30(-0.41,-0.20) |
| Chad | 309.36(83.23,600.26) | 12.28(1.28,26.69) | 360.51(105.25,676.17) | 14.67(1.99,30.76) | 0.42(0.16,0.67) | 0.51(0.25,0.77) |
| Chile | 160.34(4.17,304.30) | 7.93(0.47,16.00) | 61.79(3.55,117.70) | 2.78(0.20,5.79) | -2.77(-2.92,-2.61) | -2.90(-3.11,-2.69) |
| China | 336.22(132.48,557.38) | 14.97(4.95,26.65) | 253.69(81.54,429.79) | 11.00(2.77,20.06) | -0.86(-0.97,-0.75) | -0.96(-1.12,-0.79) |
| Colombia | 124.29(39.25,215.35) | 6.09(1.58,11.26) | 49.09(14.57,85.95) | 2.30(0.61,4.33) | -3.57(-3.91,-3.24) | -3.72(-4.01,-3.42) |
| Comoros | 271.78(100.86,469.14) | 11.79(3.02,22.18) | 175.63(59.23,310.82) | 7.50(1.37,14.77) | -1.73(-1.93,-1.52) | -1.81(-2.04,-1.59) |
| Congo | 238.81(86.84,431.80) | 9.86(1.34,20.43) | 191.15(62.86,352.01) | 8.24(1.09,17.11) | -1.05(-1.20,-0.89) | -0.92(-1.08,-0.75) |
| Cook Islands | 167.73(50.08,292.16) | 7.36(1.65,13.94) | 95.53(22.32,173.96) | 3.53(0.55,6.98) | -1.93(-2.11,-1.76) | -2.53(-2.74,-2.32) |
| Costa Rica | 79.09(20.06,143.04) | 3.75(0.57,7.56) | 41.07(7.31,76.86) | 1.93(0.20,3.99) | -2.58(-2.98,-2.17) | -2.67(-3.15,-2.19) |
| Croatia | 523.58(189.59,839.94) | 30.26(10.36,50.34) | 164.11(57.46,281.90) | 9.12(2.62,15.90) | -3.92(-4.04,-3.81) | -4.08(-4.21,-3.94) |
| Cuba | 86.60(4.56,170.52) | 4.39(0.22,9.25) | 55.27(0.12,118.73) | 2.78(0.06,6.60) | -1.59(-1.75,-1.42) | -1.66(-1.81,-1.51) |
| Cyprus | 127.62(-16.67,314.66) | 8.81(-2.10,23.37) | 36.86(-3.12,82.77) | 2.36(-0.54,5.83) | -4.33(-4.56,-4.10) | -4.51(-4.85,-4.18) |
| Czechia | 627.25(267.97,999.04) | 34.62(13.58,56.78) | 124.30(43.87,204.49) | 5.93(1.90,10.29) | -5.55(-5.81,-5.28) | -6.11(-6.52,-5.70) |
| C么te d'Ivoire | 223.20(30.58,435.28) | 9.20(0.66,19.66) | 199.42(31.01,406.39) | 8.36(0.76,18.57) | -0.49(-0.71,-0.26) | -0.39(-0.61,-0.17) |
| Democratic People's Republic of Korea | 308.67(118.29,543.70) | 12.79(4.05,24.36) | 285.07(100.83,488.15) | 11.55(3.46,21.28) | -0.33(-0.51,-0.16) | -0.38(-0.56,-0.20) |
| Democratic Republic of the Congo | 162.88(61.55,291.67) | 6.57(1.10,13.33) | 153.14(49.55,304.84) | 6.30(0.70,14.31) | -0.30(-0.40,-0.20) | -0.24(-0.35,-0.14) |
| Denmark | 99.65(10.10,195.68) | 4.59(0.48,9.91) | 39.46(-1.73,78.91) | 1.75(0.06,3.93) | -3.27(-3.41,-3.12) | -3.44(-3.63,-3.25) |
| Djibouti | 262.80(83.33,463.93) | 11.15(2.30,21.40) | 206.13(52.70,387.44) | 8.74(1.26,17.84) | -1.06(-1.17,-0.95) | -1.08(-1.20,-0.96) |
| Dominica | 120.82(-22.92,266.53) | 6.66(-0.97,15.40) | 93.77(-19.44,213.68) | 4.95(-0.87,12.04) | -0.89(-1.06,-0.71) | -1.08(-1.22,-0.93) |
| Dominican Republic | 89.39(10.30,168.34) | 4.35(0.33,8.99) | 66.14(3.33,141.34) | 3.00(0.20,7.11) | -0.83(-0.96,-0.69) | -0.92(-1.13,-0.71) |
| Ecuador | 119.02(27.11,201.50) | 5.23(0.86,9.81) | 48.33(8.33,91.30) | 2.29(0.18,4.86) | -2.93(-3.24,-2.62) | -2.59(-2.90,-2.28) |
| Egypt | 217.70(-73.08,546.33) | 9.57(-2.89,25.57) | 148.81(-52.28,371.92) | 6.30(-2.06,17.25) | -0.90(-1.05,-0.75) | -0.98(-1.13,-0.82) |
| El Salvador | 85.05(26.64,143.76) | 3.70(0.84,6.89) | 42.77(9.39,81.60) | 1.93(0.30,4.11) | -2.49(-2.94,-2.04) | -2.38(-2.81,-1.95) |
| Equatorial Guinea | 222.03(76.64,416.44) | 9.12(1.36,18.90) | 121.95(36.59,248.71) | 5.57(0.76,13.09) | -2.56(-3.00,-2.13) | -2.19(-2.60,-1.77) |
| Eritrea | 266.52(101.83,487.28) | 11.37(3.11,22.38) | 188.71(61.59,350.01) | 8.28(1.58,17.11) | -1.18(-1.30,-1.06) | -1.11(-1.24,-0.99) |
| Estonia | 318.36(-15.32,629.92) | 13.93(-0.10,29.28) | 62.87(1.33,120.95) | 2.35(0.22,4.89) | -6.71(-7.29,-6.13) | -7.36(-8.03,-6.68) |
| Eswatini | 155.85(46.70,297.84) | 6.87(0.61,14.97) | 153.62(57.23,292.53) | 6.61(0.75,14.25) | 0.39(-0.09,0.88) | 0.38(-0.10,0.87) |
| Ethiopia | 200.36(77.61,374.12) | 8.02(2.01,16.14) | 120.86(40.41,219.33) | 5.01(0.71,10.15) | -1.99(-2.13,-1.86) | -1.89(-2.03,-1.76) |
| Fiji | 238.62(91.03,372.51) | 9.22(2.51,16.02) | 171.72(47.97,302.41) | 7.01(1.41,13.63) | -1.37(-1.55,-1.19) | -1.32(-1.56,-1.08) |
| Finland | 144.35(13.49,267.93) | 6.34(0.66,12.64) | 43.58(2.76,85.46) | 1.64(0.06,3.64) | -3.75(-3.97,-3.53) | -4.27(-4.48,-4.07) |
| France | 86.74(-12.60,182.55) | 4.51(-0.57,10.49) | 37.92(-4.24,74.61) | 1.39(-0.08,3.06) | -2.54(-2.65,-2.42) | -3.69(-3.84,-3.54) |
| Gabon | 141.56(32.23,272.46) | 5.78(0.79,12.34) | 116.04(33.52,232.24) | 5.20(0.66,11.81) | -0.81(-0.95,-0.68) | -0.53(-0.71,-0.35) |
| Gambia | 364.51(160.30,616.48) | 14.15(3.85,27.07) | 371.59(150.60,640.88) | 15.20(3.19,29.36) | -0.03(-0.19,0.12) | 0.18(0.04,0.31) |
| Georgia | 239.27(12.79,459.94) | 12.14(1.38,24.07) | 258.68(-11.43,503.66) | 12.84(0.49,26.34) | 0.12(-0.33,0.57) | 0.12(-0.54,0.78) |
| Germany | 127.89(13.47,254.56) | 6.05(0.74,13.47) | 47.97(2.97,90.60) | 1.61(0.14,3.56) | -3.10(-3.40,-2.81) | -4.25(-4.54,-3.95) |
| Ghana | 291.44(25.66,559.02) | 11.71(0.85,24.51) | 279.22(29.00,543.73) | 11.96(1.36,25.38) | 0.23(-0.02,0.47) | 0.48(0.19,0.77) |
| Greece | 144.15(-38.09,330.25) | 8.61(-1.79,21.47) | 50.31(-10.99,111.04) | 2.44(-0.40,5.83) | -3.91(-4.16,-3.66) | -4.73(-5.10,-4.36) |
| Greenland | 178.87(-1.64,379.19) | 8.05(-0.06,19.20) | 69.85(-0.57,145.68) | 2.90(0.05,6.84) | -3.20(-3.32,-3.08) | -3.45(-3.61,-3.28) |
| Grenada | 293.06(34.03,538.75) | 13.33(1.43,26.41) | 115.00(6.23,237.29) | 5.66(-0.29,12.75) | -2.94(-3.09,-2.78) | -2.76(-2.96,-2.55) |
| Guam | 151.96(38.34,264.88) | 6.75(1.36,12.95) | 81.20(16.37,142.20) | 2.07(0.43,3.63) | -1.98(-2.25,-1.70) | -3.57(-4.01,-3.11) |
| Guatemala | 76.03(20.45,141.18) | 3.80(0.69,7.82) | 41.76(7.32,79.19) | 2.09(0.24,4.44) | -2.47(-2.81,-2.14) | -2.48(-2.85,-2.12) |
| Guinea | 146.96(25.05,304.36) | 6.20(0.57,14.75) | 179.39(42.88,355.78) | 7.59(1.14,16.70) | 0.98(0.78,1.19) | 1.02(0.81,1.24) |
| Guinea-Bissau | 370.60(171.70,648.39) | 14.58(4.43,28.11) | 335.34(141.16,599.94) | 13.69(3.47,27.06) | -0.15(-0.23,-0.06) | 0.01(-0.08,0.10) |
| Guyana | 347.72(115.73,610.01) | 15.70(4.00,29.75) | 146.56(22.72,298.40) | 7.12(0.65,15.81) | -2.21(-2.45,-1.97) | -2.01(-2.25,-1.77) |
| Haiti | 313.04(72.33,569.73) | 14.94(1.88,29.40) | 233.38(63.20,477.88) | 11.05(1.08,25.15) | -0.87(-0.92,-0.82) | -0.91(-0.95,-0.88) |
| Honduras | 125.06(38.04,218.64) | 5.81(1.28,11.06) | 123.87(29.77,242.72) | 6.57(0.93,13.81) | 0.17(-0.02,0.37) | 0.61(0.39,0.84) |
| Hungary | 624.72(261.49,940.78) | 30.27(10.98,47.10) | 199.62(70.05,326.28) | 9.02(3.02,15.40) | -4.10(-4.31,-3.89) | -4.30(-4.53,-4.08) |
| Iceland | 119.40(1.59,226.27) | 5.33(-0.11,10.90) | 35.28(0.76,68.41) | 1.36(-0.03,2.97) | -4.02(-4.17,-3.87) | -4.44(-4.61,-4.28) |
| India | 154.43(63.38,269.27) | 6.22(1.66,11.87) | 119.68(43.96,210.19) | 4.82(1.04,9.25) | -0.98(-1.07,-0.89) | -0.96(-1.07,-0.86) |
| Indonesia | 344.72(159.87,533.17) | 14.30(4.68,24.24) | 302.28(97.14,568.49) | 14.25(3.39,28.93) | -0.42(-0.49,-0.35) | 0.01(-0.09,0.11) |
| Iran (Islamic Republic of) | 198.48(-96.33,456.64) | 7.62(-3.18,18.86) | 92.82(-56.88,217.75) | 3.45(-1.90,8.98) | -2.55(-2.68,-2.41) | -2.68(-2.83,-2.52) |
| Iraq | 286.88(-132.21,651.39) | 10.86(-4.14,26.71) | 218.62(-91.33,505.34) | 8.89(-3.32,22.68) | -1.47(-1.65,-1.29) | -1.31(-1.52,-1.11) |
| Ireland | 107.72(10.99,208.89) | 5.36(0.24,11.32) | 24.26(-0.04,48.98) | 1.07(-0.01,2.47) | -4.97(-5.22,-4.72) | -5.16(-5.42,-4.90) |
| Israel | 64.23(-15.77,143.21) | 2.99(-0.55,7.26) | 23.22(-5.49,51.16) | 0.81(-0.13,2.04) | -3.69(-3.89,-3.49) | -4.72(-4.93,-4.51) |
| Italy | 130.26(-14.44,264.56) | 7.21(-0.59,15.52) | 38.14(-3.68,77.01) | 1.91(-0.14,4.23) | -4.19(-4.54,-3.83) | -4.48(-4.79,-4.17) |
| Jamaica | 127.55(3.52,261.84) | 6.74(0.09,15.05) | 82.28(-1.54,181.51) | 4.31(-0.11,10.48) | -1.18(-1.62,-0.73) | -1.28(-1.73,-0.83) |
| Japan | 177.88(58.82,297.43) | 9.39(2.58,16.84) | 51.92(13.63,91.78) | 1.86(0.31,3.68) | -4.36(-4.57,-4.15) | -5.63(-5.86,-5.40) |
| Jordan | 232.38(-93.40,516.33) | 8.99(-3.42,22.24) | 107.30(-41.59,235.98) | 3.91(-1.45,10.14) | -3.06(-3.42,-2.71) | -3.31(-3.71,-2.90) |
| Kazakhstan | 547.94(29.43,940.50) | 23.12(3.37,41.41) | 295.64(-44.10,599.19) | 13.76(-0.61,29.18) | -2.74(-3.21,-2.26) | -2.35(-2.80,-1.90) |
| Kenya | 99.41(28.51,182.07) | 4.20(0.69,8.63) | 91.60(22.21,176.12) | 4.11(0.51,8.80) | -0.04(-0.15,0.06) | 0.24(0.10,0.39) |
| Kiribati | 245.22(67.33,404.44) | 9.53(2.08,17.14) | 232.43(54.29,394.21) | 9.22(1.57,17.22) | -0.29(-0.35,-0.23) | -0.22(-0.28,-0.16) |
| Kuwait | 79.00(-18.16,170.50) | 2.78(-0.43,6.55) | 56.66(-9.07,121.70) | 2.00(-0.22,4.75) | -0.86(-1.74,0.03) | -0.79(-1.95,0.39) |
| Kyrgyzstan | 455.11(24.41,828.41) | 20.35(2.33,37.55) | 229.18(-31.00,440.68) | 8.96(-0.34,18.37) | -2.98(-3.33,-2.63) | -3.30(-3.64,-2.95) |
| Lao People's Democratic Republic | 543.88(263.78,883.88) | 23.48(9.07,40.75) | 227.15(69.39,424.27) | 10.93(2.25,22.05) | -3.14(-3.39,-2.90) | -2.77(-3.00,-2.54) |
| Latvia | 400.33(-6.62,773.70) | 18.54(0.67,38.57) | 214.65(19.22,409.44) | 10.05(0.98,20.66) | -2.60(-2.89,-2.31) | -2.56(-2.87,-2.25) |
| Lebanon | 122.51(-100.01,314.93) | 4.72(-3.31,13.77) | 50.04(-33.37,117.86) | 1.59(-0.89,4.43) | -2.92(-3.26,-2.57) | -3.62(-3.94,-3.30) |
| Lesotho | 143.27(47.55,259.26) | 6.36(0.34,13.02) | 228.18(83.57,401.69) | 9.99(1.55,19.57) | 2.38(1.87,2.89) | 2.44(1.89,2.99) |
| Liberia | 206.79(65.98,379.10) | 8.64(1.33,17.75) | 223.32(71.30,432.22) | 9.35(1.54,19.72) | 0.01(-0.18,0.20) | 0.04(-0.16,0.23) |
| Libya | 101.46(-50.76,228.44) | 3.71(-1.52,9.65) | 126.99(-58.36,282.92) | 4.45(-1.70,10.90) | 0.98(0.81,1.15) | 0.92(0.71,1.13) |
| Lithuania | 269.57(-22.77,511.00) | 10.96(0.20,21.60) | 153.59(3.49,290.03) | 6.77(0.74,13.74) | -1.79(-2.11,-1.47) | -1.84(-2.17,-1.51) |
| Luxembourg | 177.89(8.33,346.34) | 9.76(0.90,20.41) | 30.39(0.21,60.28) | 1.34(0.00,3.06) | -5.80(-5.94,-5.67) | -6.26(-6.36,-6.17) |
| Madagascar | 290.40(115.85,497.52) | 12.86(3.57,23.51) | 253.59(92.83,452.01) | 10.93(2.63,21.50) | -0.59(-0.66,-0.51) | -0.70(-0.79,-0.61) |
| Malawi | 210.56(79.44,374.89) | 9.61(2.68,18.49) | 198.37(66.02,369.48) | 9.18(2.10,18.91) | -0.56(-0.80,-0.32) | -0.50(-0.75,-0.25) |
| Malaysia | 279.84(114.58,444.27) | 11.17(3.86,18.91) | 161.12(44.79,280.91) | 6.55(1.35,12.36) | -1.69(-1.86,-1.51) | -1.59(-1.76,-1.43) |
| Maldives | 390.60(132.31,638.86) | 16.44(4.24,29.07) | 127.39(24.20,225.73) | 5.90(0.79,11.46) | -4.13(-4.33,-3.93) | -3.77(-3.93,-3.61) |
| Mali | 191.09(48.56,379.04) | 7.73(0.64,17.04) | 146.93(42.05,285.43) | 6.21(0.93,13.58) | -0.73(-0.93,-0.53) | -0.52(-0.71,-0.32) |
| Malta | 131.69(-6.05,261.39) | 6.67(-0.03,14.02) | 31.73(-2.68,65.54) | 1.41(-0.10,3.28) | -4.73(-4.93,-4.53) | -5.02(-5.33,-4.72) |
| Marshall Islands | 267.76(85.72,467.01) | 11.64(3.14,21.64) | 222.33(63.00,399.73) | 9.25(1.92,18.23) | -0.68(-0.74,-0.61) | -0.84(-0.90,-0.78) |
| Mauritania | 411.74(137.08,740.07) | 16.07(2.85,32.29) | 272.47(72.81,506.96) | 11.39(0.79,23.66) | -1.54(-1.70,-1.37) | -1.30(-1.48,-1.12) |
| Mauritius | 565.07(245.31,857.77) | 23.17(8.18,37.70) | 147.22(43.94,243.52) | 5.88(1.33,10.70) | -5.43(-6.01,-4.86) | -5.67(-6.29,-5.05) |
| Mexico | 75.46(19.65,146.95) | 3.76(0.61,8.20) | 36.52(7.72,70.68) | 1.60(0.26,3.50) | -2.42(-2.62,-2.21) | -2.78(-2.97,-2.59) |
| Micronesia (Federated States of) | 296.85(96.91,495.46) | 12.51(3.27,23.29) | 233.48(64.17,412.58) | 9.47(1.88,18.53) | -0.88(-0.97,-0.80) | -1.03(-1.13,-0.93) |
| Monaco | 164.26(-10.71,327.46) | 8.88(0.20,18.58) | 71.69(-3.95,142.90) | 3.57(0.14,7.64) | -2.77(-2.95,-2.59) | -3.04(-3.27,-2.82) |
| Mongolia | 197.35(57.94,316.53) | 6.58(1.45,11.87) | 162.00(14.51,276.07) | 5.19(0.71,9.90) | -0.79(-1.09,-0.49) | -0.99(-1.38,-0.59) |
| Montenegro | 168.65(55.83,289.66) | 8.69(2.89,14.96) | 199.45(54.62,349.47) | 12.29(3.07,22.13) | 0.69(0.60,0.77) | 1.39(1.14,1.65) |
| Morocco | 225.05(-81.36,468.01) | 8.62(-2.49,20.60) | 171.35(-95.35,410.98) | 6.72(-3.24,17.13) | -0.82(-0.88,-0.76) | -0.71(-0.79,-0.64) |
| Mozambique | 374.87(141.86,630.83) | 16.34(4.58,29.64) | 380.37(127.61,664.90) | 16.30(3.85,31.30) | 0.32(0.15,0.49) | 0.28(0.13,0.43) |
| Myanmar | 525.94(251.01,824.18) | 22.62(8.28,37.64) | 238.47(79.64,432.75) | 11.14(2.75,21.79) | -2.90(-3.02,-2.78) | -2.63(-2.75,-2.51) |
| Namibia | 282.09(81.07,495.86) | 12.00(1.07,23.77) | 168.45(40.72,311.30) | 7.57(0.08,15.95) | -2.21(-2.52,-1.89) | -2.00(-2.32,-1.67) |
| Nauru | 379.18(105.67,629.34) | 16.12(3.82,29.43) | 332.69(87.59,577.23) | 13.41(2.87,25.87) | -0.63(-1.07,-0.19) | -0.80(-1.20,-0.39) |
| Nepal | 161.03(61.47,297.39) | 7.08(1.46,14.38) | 91.99(21.21,185.97) | 4.50(0.67,9.46) | -1.73(-1.94,-1.52) | -1.34(-1.56,-1.13) |
| Netherlands | 81.91(3.31,167.11) | 3.71(0.05,8.43) | 39.95(-0.42,79.96) | 1.71(-0.08,3.93) | -2.67(-2.90,-2.45) | -2.99(-3.31,-2.68) |
| New Zealand | 90.20(-0.90,177.86) | 4.25(0.20,9.21) | 38.80(-2.17,77.55) | 1.63(-0.03,3.61) | -2.85(-3.03,-2.67) | -3.29(-3.46,-3.12) |
| Nicaragua | 110.21(39.33,179.86) | 4.86(1.11,8.83) | 64.20(25.44,108.08) | 2.70(0.60,5.21) | -1.79(-1.95,-1.63) | -1.84(-2.05,-1.63) |
| Niger | 200.69(67.80,390.76) | 8.03(1.09,18.06) | 146.28(37.40,296.95) | 6.39(0.40,14.50) | -1.07(-1.21,-0.93) | -0.71(-0.82,-0.60) |
| Nigeria | 171.15(18.74,366.52) | 7.40(0.38,17.03) | 116.19(9.31,238.94) | 5.00(0.22,11.57) | -1.41(-1.56,-1.25) | -1.41(-1.57,-1.25) |
| Niue | 234.51(68.26,406.95) | 10.12(2.19,18.75) | 178.85(44.16,305.63) | 7.54(1.30,14.46) | -1.19(-1.29,-1.09) | -1.27(-1.37,-1.17) |
| North Macedonia | 810.05(285.47,1315.74) | 44.55(14.78,74.29) | 621.31(155.56,1092.57) | 40.76(10.37,72.80) | -1.18(-1.52,-0.84) | -0.64(-1.12,-0.17) |
| Northern Mariana Islands | 174.05(47.99,313.03) | 8.01(1.71,14.78) | 118.08(27.69,208.97) | 5.01(0.86,9.86) | -1.57(-1.79,-1.34) | -1.96(-2.25,-1.67) |
| Norway | 143.43(13.56,267.02) | 7.02(0.80,13.87) | 44.35(3.07,82.05) | 1.69(0.12,3.40) | -4.05(-4.16,-3.95) | -4.81(-5.01,-4.62) |
| Oman | 154.58(-59.26,350.09) | 5.86(-2.07,14.28) | 77.70(-26.15,179.88) | 2.98(-0.90,7.71) | -1.89(-2.07,-1.70) | -1.74(-2.02,-1.45) |
| Pakistan | 172.28(24.83,343.20) | 7.17(0.54,15.37) | 179.60(22.61,340.16) | 7.64(0.95,15.86) | 0.02(-0.12,0.16) | 0.08(-0.07,0.24) |
| Palau | 270.15(79.53,466.16) | 11.60(2.73,21.37) | 216.40(46.42,371.02) | 8.96(1.72,17.35) | -0.67(-0.72,-0.61) | -0.74(-0.82,-0.65) |
| Palestine | 331.99(1.66,686.99) | 14.48(-0.81,34.17) | 132.32(-49.22,305.36) | 5.83(-2.17,15.50) | -2.93(-3.08,-2.78) | -2.88(-3.05,-2.72) |
| Panama | 98.72(27.90,179.72) | 4.89(0.90,9.76) | 52.43(13.96,100.36) | 2.68(0.38,5.76) | -2.30(-2.50,-2.09) | -2.18(-2.41,-1.95) |
| Papua New Guinea | 170.79(37.21,322.68) | 7.91(1.44,15.95) | 143.57(28.83,270.16) | 6.57(0.82,13.37) | -0.67(-0.78,-0.57) | -0.72(-0.83,-0.62) |
| Paraguay | 155.89(20.22,287.34) | 7.81(0.88,15.48) | 92.25(14.74,178.09) | 4.61(0.55,9.70) | -1.80(-2.01,-1.59) | -1.63(-1.88,-1.38) |
| Peru | 70.02(15.84,128.85) | 3.27(0.36,6.67) | 36.51(6.65,72.51) | 1.62(0.19,3.48) | -2.68(-3.17,-2.20) | -2.91(-3.44,-2.37) |
| Philippines | 191.38(71.07,318.50) | 9.12(2.56,16.11) | 155.99(50.42,279.95) | 6.65(1.68,12.73) | -0.68(-0.74,-0.61) | -0.95(-1.03,-0.87) |
| Poland | 412.34(70.69,728.31) | 23.23(4.68,42.39) | 147.11(26.82,255.09) | 7.43(1.47,13.51) | -3.61(-3.78,-3.44) | -4.03(-4.17,-3.88) |
| Portugal | 176.50(-12.09,392.29) | 9.84(-0.94,24.64) | 42.49(-6.24,89.99) | 2.03(-0.25,4.79) | -4.98(-5.21,-4.75) | -5.56(-5.80,-5.32) |
| Puerto Rico | 73.49(16.51,132.60) | 3.80(-0.03,7.87) | 26.02(4.15,48.79) | 1.02(0.02,2.18) | -3.91(-4.14,-3.68) | -4.80(-5.07,-4.54) |
| Qatar | 112.62(-52.61,277.21) | 4.80(-2.17,13.49) | 47.31(-18.67,108.95) | 1.97(-0.63,5.27) | -3.11(-3.67,-2.54) | -3.34(-4.10,-2.58) |
| Republic of Korea | 348.23(96.93,637.99) | 19.00(4.74,35.73) | 85.03(24.95,149.88) | 4.12(0.98,7.65) | -5.14(-5.39,-4.90) | -5.61(-5.88,-5.33) |
| Republic of Moldova | 135.34(-4.74,291.95) | 6.50(-0.07,15.14) | 119.03(6.36,235.84) | 4.65(0.31,10.03) | -0.27(-0.81,0.27) | -0.92(-1.44,-0.40) |
| Romania | 591.59(219.85,967.52) | 34.27(11.70,57.65) | 336.71(109.94,550.87) | 18.59(6.15,30.92) | -2.35(-2.58,-2.11) | -2.45(-2.68,-2.21) |
| Russian Federation | 578.83(37.60,1057.90) | 26.50(2.13,52.20) | 269.08(19.11,493.53) | 11.96(1.15,23.85) | -3.55(-4.14,-2.96) | -3.64(-4.25,-3.02) |
| Rwanda | 350.39(109.33,612.97) | 15.54(3.82,29.17) | 130.89(21.79,259.81) | 6.22(0.67,13.57) | -4.30(-4.79,-3.82) | -4.08(-4.57,-3.58) |
| Saint Kitts and Nevis | 343.05(69.51,621.86) | 17.13(1.34,34.50) | 147.68(14.62,299.61) | 7.60(-0.11,17.02) | -2.58(-2.88,-2.29) | -2.45(-2.69,-2.22) |
| Saint Lucia | 262.73(28.61,508.23) | 14.25(-0.66,30.05) | 102.31(7.26,216.49) | 5.43(-0.35,12.75) | -3.41(-3.82,-3.00) | -3.77(-4.24,-3.30) |
| Saint Vincent and the Grenadines | 195.93(30.97,370.78) | 9.91(0.10,20.83) | 94.58(6.59,190.23) | 4.89(-0.12,11.00) | -2.45(-2.70,-2.20) | -2.30(-2.56,-2.04) |
| Samoa | 182.89(49.01,326.96) | 7.03(0.33,14.42) | 164.03(35.80,284.13) | 6.26(0.46,12.37) | -0.49(-0.58,-0.40) | -0.57(-0.68,-0.45) |
| San Marino | 95.22(-1.47,192.01) | 4.95(-0.08,10.97) | 36.61(-2.40,76.89) | 1.46(0.01,3.43) | -2.72(-2.96,-2.48) | -3.31(-3.68,-2.94) |
| Sao Tome and Principe | 220.81(39.80,407.09) | 8.38(0.63,17.43) | 223.59(12.76,418.80) | 8.86(0.28,18.83) | 0.05(-0.05,0.16) | 0.29(0.19,0.39) |
| Saudi Arabia | 163.74(-48.98,370.01) | 6.62(-1.64,16.87) | 132.67(-27.41,276.53) | 4.97(-0.72,11.62) | -0.71(-0.88,-0.53) | -1.06(-1.24,-0.88) |
| Senegal | 293.43(78.50,534.70) | 11.57(1.87,23.26) | 205.33(54.40,407.54) | 8.76(0.92,19.26) | -1.15(-1.34,-0.96) | -0.90(-1.07,-0.73) |
| Serbia | 838.51(304.43,1363.30) | 50.41(16.68,83.95) | 414.61(128.75,723.89) | 24.56(7.04,44.31) | -2.84(-3.09,-2.59) | -2.92(-3.19,-2.65) |
| Seychelles | 289.77(125.70,475.73) | 12.20(3.94,21.42) | 152.92(49.99,265.77) | 6.58(1.55,12.60) | -1.88(-2.05,-1.70) | -1.72(-1.96,-1.48) |
| Sierra Leone | 248.68(76.05,465.91) | 9.98(1.92,21.03) | 220.75(61.49,412.58) | 9.01(1.57,18.65) | -0.20(-0.43,0.03) | -0.13(-0.38,0.12) |
| Singapore | 196.35(53.40,351.19) | 8.54(1.95,15.90) | 33.42(7.92,62.09) | 0.89(0.15,1.83) | -5.90(-6.05,-5.74) | -7.19(-7.64,-6.73) |
| Slovakia | 473.17(194.80,757.45) | 22.87(8.50,37.54) | 220.77(70.72,371.29) | 10.36(3.20,17.65) | -2.59(-2.68,-2.49) | -2.70(-2.79,-2.60) |
| Slovenia | 377.86(157.14,577.11) | 20.03(7.55,31.23) | 99.14(36.01,162.22) | 5.36(1.80,8.91) | -4.41(-4.66,-4.16) | -4.27(-4.53,-4.02) |
| Solomon Islands | 275.93(102.96,460.43) | 11.48(2.96,21.20) | 242.62(88.59,434.43) | 9.77(2.07,19.88) | -0.50(-0.66,-0.34) | -0.62(-0.79,-0.44) |
| Somalia | 246.81(92.60,472.87) | 10.46(2.73,21.74) | 197.93(74.08,369.19) | 8.10(1.91,16.79) | -0.76(-0.82,-0.71) | -0.85(-0.91,-0.80) |
| South Africa | 103.62(40.55,178.33) | 3.54(0.47,7.28) | 103.93(33.79,190.29) | 4.26(0.34,9.06) | 0.03(-0.42,0.49) | 0.64(0.07,1.21) |
| South Sudan | 185.16(56.58,346.99) | 8.40(1.80,16.67) | 137.97(38.92,270.34) | 6.11(1.25,12.58) | -1.26(-1.51,-1.01) | -1.35(-1.61,-1.09) |
| Spain | 84.75(-20.03,183.55) | 4.36(-0.81,10.47) | 24.69(-3.47,51.43) | 0.81(-0.09,1.95) | -4.13(-4.54,-3.71) | -5.62(-6.01,-5.23) |
| Sri Lanka | 361.14(124.72,590.05) | 18.59(4.56,33.52) | 193.86(51.95,359.11) | 9.96(1.66,20.42) | -1.74(-1.95,-1.52) | -1.48(-1.74,-1.22) |
| Sudan | 257.83(-57.24,565.16) | 9.87(-1.85,23.73) | 187.78(-58.83,425.98) | 7.01(-1.80,17.88) | -1.18(-1.25,-1.10) | -1.29(-1.37,-1.22) |
| Suriname | 123.58(26.43,241.36) | 6.00(0.77,12.87) | 86.86(16.74,175.84) | 4.01(0.47,8.87) | -1.24(-1.56,-0.93) | -1.35(-1.67,-1.02) |
| Sweden | 99.45(6.26,195.15) | 4.80(0.13,10.09) | 40.28(0.67,79.53) | 1.54(0.01,3.43) | -3.13(-3.27,-3.00) | -3.81(-4.07,-3.55) |
| Switzerland | 78.66(-4.61,163.24) | 3.96(-0.32,9.14) | 25.70(-2.80,52.87) | 0.96(-0.13,2.26) | -3.51(-3.71,-3.31) | -4.39(-4.56,-4.21) |
| Syrian Arab Republic | 164.82(-85.36,359.60) | 6.02(-2.59,15.41) | 128.77(-47.95,286.96) | 4.94(-1.74,12.20) | -1.30(-1.55,-1.05) | -1.21(-1.47,-0.95) |
| Taiwan (Province of China) | 190.70(44.36,344.30) | 8.84(1.68,16.52) | 65.08(3.40,122.47) | 2.06(0.25,3.98) | -3.38(-3.57,-3.19) | -4.65(-4.91,-4.38) |
| Tajikistan | 337.59(18.44,626.80) | 15.01(2.07,28.38) | 216.69(-14.13,438.23) | 10.17(0.14,21.05) | -1.89(-2.27,-1.52) | -1.71(-2.15,-1.26) |
| Thailand | 196.56(80.79,317.89) | 8.07(2.47,14.26) | 98.74(32.36,176.95) | 3.80(0.91,7.82) | -2.77(-2.98,-2.57) | -3.08(-3.33,-2.84) |
| Timor-Leste | 307.23(137.16,486.13) | 13.77(4.62,23.82) | 293.64(112.86,493.15) | 13.29(3.47,24.59) | -0.07(-0.22,0.08) | 0.01(-0.16,0.17) |
| Togo | 344.08(120.75,592.92) | 13.57(2.87,26.69) | 341.56(142.30,574.79) | 13.74(3.31,26.47) | -0.07(-0.29,0.15) | -0.01(-0.23,0.21) |
| Tokelau | 253.65(74.28,432.76) | 11.47(2.73,21.47) | 165.30(45.05,292.74) | 6.94(1.19,13.63) | -1.59(-1.66,-1.51) | -1.82(-1.90,-1.75) |
| Tonga | 143.47(49.38,232.56) | 5.79(1.32,10.40) | 119.72(32.86,212.91) | 4.83(0.82,9.72) | -0.59(-0.70,-0.48) | -0.53(-0.68,-0.39) |
| Trinidad and Tobago | 210.16(34.74,386.56) | 9.83(0.68,20.35) | 102.09(7.28,203.41) | 4.64(0.05,10.40) | -2.95(-3.25,-2.65) | -2.94(-3.23,-2.65) |
| Tunisia | 128.81(-48.33,297.34) | 5.39(-2.03,13.87) | 92.21(-49.57,215.34) | 3.67(-1.62,10.05) | -1.28(-1.37,-1.19) | -1.46(-1.56,-1.36) |
| Turkey | 133.46(-99.25,326.16) | 5.20(-3.36,14.20) | 61.11(-41.38,157.53) | 2.47(-1.42,7.16) | -2.78(-2.96,-2.59) | -2.52(-2.79,-2.25) |
| Turkmenistan | 336.43(-19.11,624.31) | 13.49(1.14,25.96) | 383.73(-96.81,769.33) | 14.73(-2.08,30.57) | 0.33(-0.11,0.78) | 0.00(-0.50,0.50) |
| Tuvalu | 310.57(110.07,517.29) | 13.48(3.30,24.87) | 224.87(65.26,373.80) | 9.44(2.09,17.54) | -1.10(-1.17,-1.03) | -1.22(-1.30,-1.15) |
| Uganda | 190.96(65.03,354.08) | 8.49(2.02,17.04) | 119.76(35.57,224.80) | 5.17(0.87,10.87) | -2.18(-2.48,-1.89) | -2.33(-2.65,-2.01) |
| Ukraine | 309.89(-62.65,659.86) | 13.71(-2.51,32.26) | 173.46(-30.31,374.28) | 6.88(-1.02,16.75) | -2.78(-3.20,-2.36) | -3.16(-3.59,-2.73) |
| United Arab Emirates | 172.57(-89.37,404.48) | 6.32(-3.07,16.27) | 94.60(-21.93,216.22) | 3.95(-1.09,10.26) | -0.74(-1.14,-0.34) | 0.25(-0.35,0.85) |
| United Kingdom | 113.79(5.45,230.03) | 5.75(0.08,12.66) | 31.05(0.97,61.36) | 1.25(-0.05,2.87) | -4.41(-4.58,-4.24) | -5.13(-5.34,-4.92) |
| United Republic of Tanzania | 152.07(53.90,265.78) | 6.88(1.77,13.27) | 161.87(46.54,302.94) | 7.76(1.52,15.75) | -0.04(-0.24,0.16) | 0.23(0.01,0.46) |
| United States of America | 71.83(2.53,134.77) | 2.82(0.32,5.92) | 56.63(5.40,102.33) | 1.77(0.22,3.63) | -1.19(-1.41,-0.96) | -2.14(-2.52,-1.76) |
| United States Virgin Islands | 83.75(4.78,170.62) | 4.38(0.12,9.60) | 36.43(0.32,75.31) | 1.74(0.00,4.01) | -2.62(-2.82,-2.42) | -2.90(-3.10,-2.71) |
| Uruguay | 254.99(12.87,458.53) | 11.66(0.53,22.57) | 103.71(1.09,198.23) | 4.61(0.15,9.53) | -3.14(-3.28,-3.00) | -3.20(-3.39,-3.01) |
| Uzbekistan | 279.92(-11.79,516.22) | 10.68(1.05,19.72) | 203.64(-41.38,418.93) | 8.29(-0.48,17.40) | -1.75(-2.08,-1.42) | -1.64(-2.04,-1.24) |
| Vanuatu | 273.91(79.01,464.34) | 11.95(2.64,21.94) | 221.51(64.67,375.17) | 8.93(1.61,16.99) | -0.90(-0.98,-0.82) | -1.18(-1.26,-1.09) |
| Venezuela (Bolivarian Republic of) | 86.41(23.38,157.84) | 4.23(0.81,8.42) | 59.00(12.76,117.68) | 3.09(0.45,6.69) | -1.68(-2.07,-1.28) | -1.37(-1.76,-0.97) |
| Viet Nam | 329.16(128.75,572.27) | 15.94(5.05,30.48) | 251.20(70.18,498.46) | 12.73(2.86,26.79) | -0.67(-0.82,-0.52) | -0.52(-0.64,-0.40) |
| Yemen | 338.54(-37.82,696.17) | 13.25(-1.33,30.17) | 305.01(-18.77,632.40) | 12.01(-1.04,27.62) | -0.67(-0.82,-0.52) | -0.66(-0.81,-0.51) |
| Zambia | 235.17(104.22,407.17) | 10.39(2.85,20.06) | 248.92(97.30,429.08) | 11.11(2.48,21.11) | -0.04(-0.17,0.09) | 0.01(-0.12,0.14) |
| Zimbabwe | 180.60(63.63,309.73) | 7.60(0.69,14.99) | 262.04(110.55,436.00) | 10.84(1.95,20.61) | 1.67(1.11,2.23) | 1.74(1.14,2.34) |

Stable2. Top Three Countries with the Highest Age-Standardized Rates (ASR) of Ischemic Stroke Attributable to Dietary Factors, 2021

| Measure | risks | Top three countries |  |  |
| --- | --- | --- | --- | --- |
| 2021 ASR (per 100,000 people) |  |  |  |  |
| Age-standardized DALY rate |  |  |  |  |
|  | Dietary risks | Serbia(414.61) | Bulgaria(571.63) | North Macedonia(621.31) |
|  | Diet low in fruits | Chad(123.72) | Togo(149.67) | Gambia(183.21) |
|  | Diet low in vegetables | Chad(143.33) | Guinea-Bissau(191.33) | Afghanistan(205.44) |
|  | Diet low in whole grains | Yemen(154.28) | Turkmenistan(174.02) | Afghanistan(175.09) |
|  | Diet high in red meat | Mongolia(33.61) | Kazakhstan(35.90) | Turkmenistan(75.06) |
|  | Diet high in processed meat | Russian Federation(32.34) | Lithuania(45.49) | Latvia(60.75) |
|  | Diet high in sugar-sweetened beverages | Saudi Arabia(9.36) | Bulgaria(11.75) | Romania(13.50) |
|  | Diet low in fiber | Lao People's Democratic Republic(57.69) | Afghanistan(63.84) | Viet Nam(65.38) |
|  | Diet low in omega-6 polyunsaturated fatty acids | Turkmenistan(0.61) | Egypt(0.63) | Afghanistan(0.64) |
|  | Diet high in sodium | Serbia(317.62) | Bulgaria(387.58) | North Macedonia(477.26) |
| ASDR | Dietary risks | Serbia(24.56) | Bulgaria(30.78) | North Macedonia(40.76) |
|  | Diet low in fruits | Chad(4.51) | Togo(5.39) | Gambia(6.77) |
|  | Diet low in vegetables | Chad(5.35) | Guinea-Bissau(7.06) | Afghanistan(7.45) |
|  | Diet low in whole grains | Yemen(5.73) | North Macedonia(6.19) | Afghanistan(6.24) |
|  | Diet high in red meat | Mongolia(0.77) | Kazakhstan(0.84) | Turkmenistan(1.97) |
|  | Diet high in processed meat | Russian Federation(1.83) | Lithuania(2.27) | Latvia(3.16) |
|  | Diet high in sugar-sweetened beverages | Bulgaria(0.60) | North Macedonia(0.62) | Romania(0.76) |
|  | Diet low in fiber | Lao People's Democratic Republic(2.03) | Afghanistan(2.18) | Viet Nam(2.46) |
|  | Diet low in omega-6 polyunsaturated fatty acids | Afghanistan(0.03) | Egypt(0.03) | North Macedonia(0.04) |
|  | Diet high in sodium | Serbia(20.01) | Bulgaria(23.30) | North Macedonia(33.13) |

stable3. Age-standardized Rate of death and DALYs for ischemic stroke attributable to dietary factors in 2021 (A. Death B. DALYs.)

|  | A | | | | | | | | | | B | | | | | | | | | |
| --- | --- | --- | --- | --- | --- | --- | --- | --- | --- | --- | --- | --- | --- | --- | --- | --- | --- | --- | --- | --- |
|  | Dietary risks | Diet low in fruits | Diet low in vegetables | Diet low in whole grains | Diet high in red meat | Diet high in processed meat | Diet high in sugar-sweetened beverages | Diet low in fiber | Diet low in omega-6 polyunsaturated fatty acids | Diet high in sodium | Dietary risks | Diet low in fruits | Diet low in vegetables | Diet low in whole grains | Diet high in red meat | Diet high in processed meat | Diet high in sugar-sweetened beverages | Diet low in fiber | Diet low in omega-6 polyunsaturated fatty acids | Diet high in sodium |
| global | 6.41(1.45,11.77) | 0.79(0.16,1.52) | 0.47(0.06,0.89) | 1.21(-1.15,3.95) | 0.00(-0.16,0.35) | 0.27(0.06,0.46) | 0.08(0.04,0.13) | 0.35(-0.03,0.89) | 0.01(0.00,0.02) | 3.96(0.77,9.23) | 153.28(45.74,259.88) | 23.75(12.40,38.17) | 13.75(7.05,21.33) | 35.95(-36.48,101.74) | 1.30(-1.64,13.25) | 5.20(1.21,9.33) | 1.72(0.83,2.73) | 10.99(-0.46,22.36) | 0.21(0.06,0.39) | 84.35(20.33,187.35) |
| High-middle SDI | 8.89(2.08,16.29) | 0.62(0.00,1.21) | 0.20(-0.03,0.43) | 1.80(-1.70,5.95) | 0.08(-0.16,0.70) | 0.50(0.11,0.88) | 0.13(0.06,0.21) | 0.29(-0.04,0.79) | 0.01(0.00,0.02) | 6.05(1.39,13.33) | 203.25(57.51,346.01) | 17.66(8.03,28.17) | 5.26(1.99,8.91) | 51.34(-51.38,142.96) | 5.47(-2.49,25.58) | 8.80(2.04,15.57) | 2.51(1.24,3.97) | 9.27(-0.31,20.00) | 0.25(0.07,0.47) | 128.48(36.38,267.84) |
| High SDI | 2.45(0.44,4.70) | 0.18(-0.04,0.40) | 0.11(-0.05,0.25) | 0.44(-0.42,1.53) | 0.06(-0.03,0.25) | 0.29(0.07,0.51) | 0.08(0.04,0.13) | 0.12(-0.06,0.36) | 0.00(0.00,0.01) | 1.42(0.20,3.61) | 66.26(11.52,114.69) | 6.97(3.45,10.87) | 3.88(1.44,6.35) | 17.01(-17.41,48.21) | 4.32(-2.76,13.75) | 7.35(1.76,13.06) | 2.15(1.04,3.44) | 4.86(-0.19,10.43) | 0.07(0.02,0.13) | 31.07(5.31,76.83) |
| Low-middle SDI | 6.71(1.29,12.69) | 1.73(0.33,3.40) | 1.07(0.05,2.15) | 1.26(-1.20,4.18) | -0.11(-0.37,0.07) | 0.13(0.03,0.24) | 0.05(0.02,0.08) | 0.62(-0.08,1.64) | 0.01(0.00,0.02) | 3.03(0.24,8.57) | 156.45(44.83,270.35) | 47.11(23.81,77.97) | 27.70(12.63,45.85) | 35.81(-36.59,103.77) | -3.70(-10.77,2.16) | 2.69(0.63,4.87) | 1.01(0.47,1.64) | 16.91(-0.76,36.08) | 0.26(0.07,0.48) | 59.66(5.77,162.79) |
| Low SDI | 6.70(1.23,13.21) | 1.53(0.26,3.11) | 1.89(0.18,3.64) | 1.42(-1.35,4.81) | -0.14(-0.44,0.06) | 0.13(0.03,0.23) | 0.02(0.01,0.04) | 0.36(-0.04,0.98) | 0.01(0.00,0.03) | 2.38(0.08,7.53) | 160.15(47.56,284.20) | 42.40(20.96,72.85) | 51.33(24.95,84.07) | 39.78(-39.57,116.69) | -4.42(-12.55,2.29) | 2.53(0.58,4.75) | 0.44(0.20,0.73) | 10.24(-0.44,22.95) | 0.27(0.08,0.53) | 44.95(1.52,140.97) |
| Middle SDI | 7.68(1.72,14.21) | 0.81(0.10,1.53) | 0.42(0.00,0.84) | 1.29(-1.21,4.16) | -0.01(-0.24,0.39) | 0.11(0.03,0.18) | 0.06(0.03,0.10) | 0.43(-0.03,1.06) | 0.01(0.00,0.02) | 5.39(1.13,11.89) | 178.49(57.12,303.52) | 23.36(11.70,37.39) | 11.70(5.43,18.47) | 37.56(-37.45,103.67) | 1.08(-3.31,14.21) | 2.21(0.53,3.89) | 1.37(0.67,2.16) | 13.33(-0.57,27.50) | 0.24(0.07,0.46) | 114.17(29.96,241.42) |
| Andean Latin America | 2.04(0.23,4.25) | 0.15(0.00,0.32) | 0.46(-0.08,0.98) | 0.39(-0.37,1.37) | -0.08(-0.27,0.04) | 0.02(0.01,0.04) | 0.04(0.02,0.07) | 0.16(-0.03,0.44) | 0.00(0.00,0.01) | 1.15(0.03,3.29) | 44.72(8.91,85.40) | 4.36(1.98,7.37) | 12.04(4.37,20.38) | 11.57(-11.45,34.13) | -2.45(-7.25,1.34) | 0.50(0.12,0.88) | 0.91(0.42,1.50) | 5.00(-0.25,10.93) | 0.06(0.02,0.12) | 20.53(0.62,59.02) |
| Australasia | 1.14(-0.02,2.54) | 0.10(-0.08,0.25) | 0.08(-0.09,0.24) | 0.20(-0.26,0.84) | 0.15(-0.10,0.55) | 0.30(0.07,0.52) | 0.10(0.05,0.16) | 0.08(-0.05,0.28) | 0.00(0.00,0.01) | 0.28(0.00,1.27) | 31.84(-2.09,62.29) | 3.56(1.16,6.23) | 2.72(0.43,5.03) | 7.75(-7.61,23.62) | 8.22(-5.30,20.82) | 5.84(1.43,10.44) | 2.00(0.99,3.16) | 3.57(-0.18,7.69) | 0.05(0.01,0.10) | 5.82(0.02,23.87) |
| Caribbean | 3.73(0.43,8.01) | 0.33(0.05,0.64) | 0.97(0.25,1.80) | 1.04(-1.01,3.44) | -0.18(-0.56,0.08) | 0.08(0.02,0.13) | 0.08(0.04,0.12) | 0.27(-0.02,0.65) | 0.01(0.00,0.01) | 1.61(0.01,5.22) | 83.96(11.94,162.61) | 9.24(4.62,14.90) | 26.83(13.86,42.12) | 28.60(-28.55,82.97) | -5.51(-15.55,2.80) | 1.53(0.37,2.63) | 1.52(0.78,2.39) | 7.94(-0.40,16.31) | 0.11(0.03,0.22) | 27.38(0.19,90.43) |
| Central Asia | 9.80(-0.42,20.07) | 0.93(0.12,1.79) | 0.16(-0.05,0.38) | 3.58(-3.64,11.87) | 0.07(-0.26,0.88) | 0.35(0.09,0.63) | 0.09(0.04,0.15) | 0.54(-0.08,1.34) | 0.02(0.00,0.03) | 5.12(0.32,13.78) | 224.80(-33.99,445.02) | 27.91(13.93,43.60) | 3.87(0.85,7.38) | 106.18(-120.30,299.35) | 6.09(-3.59,33.84) | 7.72(1.84,13.83) | 2.08(1.02,3.22) | 16.61(-0.77,34.62) | 0.35(0.10,0.66) | 88.81(4.77,247.92) |
| Central Europe | 12.92(3.76,22.03) | 0.62(-0.07,1.30) | 0.21(-0.08,0.48) | 1.75(-1.67,6.34) | 0.09(-0.22,0.78) | 0.74(0.19,1.28) | 0.34(0.17,0.55) | 0.36(-0.10,0.99) | 0.01(0.00,0.02) | 9.86(2.62,18.93) | 243.76(73.34,405.06) | 16.53(6.92,28.07) | 4.96(1.00,9.31) | 46.06(-46.02,137.94) | 6.02(-3.86,25.64) | 13.65(3.38,24.23) | 6.36(3.11,10.13) | 9.51(-0.57,21.78) | 0.22(0.06,0.42) | 169.10(46.75,324.95) |
| Central Latin America | 2.11(0.42,4.23) | 0.17(0.00,0.34) | 0.32(-0.04,0.67) | 0.29(-0.26,1.00) | -0.07(-0.25,0.06) | 0.05(0.01,0.08) | 0.06(0.03,0.09) | 0.10(-0.02,0.28) | 0.00(0.00,0.01) | 1.38(0.12,3.62) | 45.40(11.59,82.89) | 4.89(2.24,7.93) | 8.75(3.49,14.34) | 8.59(-7.81,24.94) | -1.88(-6.07,2.05) | 1.00(0.24,1.77) | 1.27(0.62,2.06) | 2.81(-0.12,6.30) | 0.07(0.02,0.13) | 25.34(2.44,65.44) |
| Central sub-Saharan Africa | 6.34(0.78,13.56) | 0.99(0.02,2.10) | 3.05(0.10,6.35) | 1.25(-1.13,4.52) | -0.12(-0.39,0.05) | 0.18(0.04,0.36) | 0.04(0.02,0.08) | 0.42(-0.08,1.22) | 0.01(0.00,0.03) | 1.33(0.00,5.97) | 150.28(45.50,286.82) | 26.60(10.51,47.73) | 78.51(30.17,135.24) | 33.62(-29.70,102.10) | -3.66(-10.91,1.89) | 3.46(0.73,6.87) | 0.80(0.34,1.44) | 11.04(-0.66,25.96) | 0.31(0.09,0.61) | 23.17(0.02,106.64) |
| East Asia | 10.81(2.72,19.68) | 0.62(-0.03,1.24) | 0.06(-0.03,0.18) | 1.68(-1.57,5.54) | 0.17(-0.11,0.90) | 0.16(0.04,0.29) | 0.08(0.04,0.12) | 0.25(-0.02,0.68) | 0.01(0.00,0.03) | 8.61(2.13,17.86) | 250.36(80.08,423.79) | 17.88(7.60,28.81) | 1.41(0.13,3.36) | 49.40(-47.23,137.99) | 9.41(-4.44,34.54) | 3.36(0.82,6.06) | 1.59(0.78,2.57) | 8.78(-0.21,19.27) | 0.29(0.08,0.55) | 187.15(59.31,369.08) |
| Eastern Europe | 10.28(0.62,21.52) | 1.30(0.06,2.57) | 0.68(0.01,1.32) | 3.25(-3.10,10.41) | -0.24(-1.01,0.56) | 1.46(0.33,2.64) | 0.14(0.07,0.23) | 0.60(-0.09,1.58) | 0.02(0.00,0.04) | 4.30(0.16,13.27) | 237.72(5.16,452.39) | 36.30(16.99,57.63) | 17.92(8.20,28.87) | 90.38(-93.82,248.92) | -5.50(-23.57,18.05) | 26.42(5.95,46.94) | 2.77(1.32,4.26) | 16.96(-0.59,36.08) | 0.37(0.10,0.72) | 89.04(4.89,257.28) |
| Eastern Sub-Saharan Africa | 7.14(1.59,13.46) | 1.18(0.06,2.32) | 1.95(0.01,3.79) | 0.97(-0.90,3.40) | -0.13(-0.42,0.06) | 0.08(0.02,0.14) | 0.02(0.01,0.03) | 0.13(-0.02,0.34) | 0.01(0.00,0.02) | 3.59(0.20,9.28) | 163.61(54.78,277.51) | 33.61(15.90,56.31) | 53.19(23.42,86.01) | 27.82(-26.69,82.12) | -4.20(-12.08,2.14) | 1.57(0.35,2.82) | 0.43(0.21,0.71) | 3.92(-0.16,8.63) | 0.25(0.08,0.49) | 67.30(3.95,177.31) |
| High-income Asia Pacific | 2.18(0.48,4.21) | 0.16(-0.05,0.36) | 0.04(-0.03,0.12) | 0.24(-0.24,0.90) | -0.05(-0.19,0.04) | 0.20(0.05,0.35) | 0.06(0.03,0.10) | 0.14(-0.05,0.41) | 0.00(0.00,0.00) | 1.60(0.21,3.69) | 57.17(15.51,100.53) | 7.54(3.76,11.80) | 1.57(0.47,2.94) | 10.43(-9.96,29.91) | -1.57(-5.85,2.71) | 5.43(1.32,9.72) | 1.45(0.71,2.35) | 6.67(-0.34,14.16) | 0.06(0.02,0.11) | 35.91(5.07,79.34) |
| High-income North America | 1.68(0.21,3.48) | 0.13(-0.05,0.31) | 0.13(-0.07,0.30) | 0.32(-0.32,1.19) | 0.07(-0.04,0.25) | 0.40(0.10,0.69) | 0.10(0.04,0.15) | 0.11(-0.06,0.34) | 0.00(0.00,0.00) | 0.65(0.01,2.25) | 54.07(5.17,97.85) | 5.90(2.90,9.37) | 5.06(1.97,8.22) | 14.23(-14.30,41.11) | 5.83(-3.75,16.40) | 11.39(2.64,20.22) | 2.84(1.36,4.48) | 4.51(-0.15,10.03) | 0.03(0.01,0.06) | 15.94(0.35,50.81) |
| North Africa and Middle East | 4.95(-1.92,12.50) | 0.59(0.11,1.11) | 0.50(0.06,0.96) | 3.13(-3.11,10.05) | -0.32(-0.98,0.15) | 0.14(0.03,0.25) | 0.13(0.06,0.22) | 0.27(-0.02,0.69) | 0.01(0.00,0.03) | 0.92(0.00,5.12) | 128.30(-51.59,290.27) | 17.42(8.72,27.76) | 14.13(6.95,22.78) | 87.36(-96.84,246.05) | -10.55(-30.02,5.54) | 2.88(0.71,5.04) | 2.83(1.37,4.53) | 8.37(-0.36,17.79) | 0.31(0.09,0.59) | 19.38(0.01,98.10) |
| Oceania | 6.52(1.06,12.81) | 0.65(0.02,1.36) | 1.19(-0.01,2.44) | 1.37(-1.25,4.82) | -0.19(-0.63,0.09) | 0.06(0.01,0.10) | 0.04(0.02,0.07) | 0.02(-0.01,0.07) | 0.01(0.00,0.02) | 3.82(0.33,9.15) | 151.30(35.91,272.21) | 20.21(8.76,34.57) | 34.40(15.43,56.48) | 41.65(-41.44,124.04) | -6.56(-19.60,3.23) | 1.16(0.28,2.02) | 0.91(0.42,1.45) | 0.63(-0.03,1.48) | 0.21(0.06,0.42) | 72.41(7.14,171.66) |
| South Asia | 5.51(1.06,10.46) | 2.10(0.39,4.12) | 0.91(0.04,1.88) | 0.88(-0.78,2.99) | -0.05(-0.16,0.02) | 0.10(0.02,0.18) | 0.04(0.02,0.07) | 0.48(-0.06,1.35) | 0.01(0.00,0.02) | 1.99(0.05,6.28) | 131.02(45.04,230.46) | 57.18(28.94,96.94) | 23.15(10.08,39.37) | 24.43(-23.14,74.19) | -1.48(-4.41,0.73) | 1.92(0.44,3.58) | 0.89(0.42,1.47) | 12.89(-0.50,29.97) | 0.20(0.06,0.39) | 39.42(1.45,118.96) |
| Southeast Asia | 9.95(2.38,19.45) | 0.93(0.05,1.80) | 1.29(-0.01,2.59) | 0.88(-0.83,2.87) | -0.23(-0.71,0.11) | 0.05(0.01,0.09) | 0.04(0.02,0.06) | 1.40(-0.13,3.40) | 0.02(0.00,0.03) | 7.41(1.07,16.80) | 219.81(74.14,396.53) | 27.00(13.08,42.59) | 35.77(15.53,57.16) | 26.32(-24.84,72.93) | -7.39(-20.16,4.02) | 1.11(0.26,1.93) | 0.83(0.40,1.29) | 41.43(-2.19,83.46) | 0.35(0.10,0.66) | 148.84(25.72,325.14) |
| Southern Latin America | 2.81(0.12,5.74) | 0.15(-0.04,0.32) | 0.19(-0.07,0.42) | 0.64(-0.63,2.32) | 0.27(-0.15,0.80) | 0.23(0.06,0.40) | 0.14(0.07,0.23) | 0.21(-0.07,0.56) | 0.00(0.00,0.01) | 1.40(0.03,3.97) | 67.05(-0.83,128.20) | 4.57(1.86,7.59) | 5.40(1.59,9.13) | 19.90(-20.20,58.62) | 11.07(-6.89,27.08) | 4.53(1.09,7.98) | 2.93(1.44,4.55) | 6.78(-0.33,14.80) | 0.07(0.02,0.14) | 26.59(0.73,74.36) |
| Southern sub-Saharan Africa | 5.19(0.63,10.44) | 1.84(-0.01,3.66) | 1.66(0.01,3.24) | 0.74(-0.65,2.54) | -0.24(-0.84,0.15) | 0.09(0.02,0.16) | 0.07(0.04,0.12) | 0.20(-0.04,0.55) | 0.01(0.00,0.02) | 1.32(0.00,5.92) | 127.26(46.81,222.41) | 50.87(22.42,82.59) | 44.01(19.14,69.14) | 20.81(-19.06,61.29) | -6.34(-20.31,4.96) | 1.81(0.45,3.16) | 1.46(0.74,2.29) | 5.77(-0.18,13.26) | 0.21(0.06,0.40) | 25.07(0.02,108.71) |
| Tropical Latin America | 3.75(0.52,7.62) | 0.22(0.01,0.43) | 0.57(-0.01,1.11) | 0.58(-0.53,1.90) | 0.35(-0.19,0.95) | 0.12(0.03,0.21) | 0.11(0.05,0.18) | 0.23(-0.03,0.60) | 0.00(0.00,0.01) | 2.04(0.08,5.79) | 81.25(11.91,151.11) | 5.84(2.69,9.39) | 14.30(6.17,22.56) | 15.67(-14.33,44.64) | 12.11(-7.16,29.51) | 2.33(0.55,4.07) | 2.14(1.01,3.35) | 6.43(-0.23,14.30) | 0.05(0.01,0.10) | 36.62(1.54,102.66) |
| Western Europe | 1.51(-0.05,3.30) | 0.11(-0.06,0.27) | 0.10(-0.07,0.25) | 0.38(-0.44,1.54) | 0.06(-0.03,0.22) | 0.27(0.06,0.47) | 0.06(0.03,0.09) | 0.10(-0.07,0.33) | 0.00(0.00,0.01) | 0.60(0.01,2.09) | 37.97(-0.51,74.33) | 3.79(1.37,6.53) | 3.15(0.66,5.48) | 12.59(-12.86,38.66) | 4.01(-2.44,10.86) | 5.13(1.18,9.29) | 1.14(0.54,1.80) | 3.50(-0.22,7.90) | 0.05(0.01,0.10) | 12.04(0.24,39.95) |
| Western sub-Saharan Africa | 7.06(0.74,14.78) | 1.37(0.15,2.64) | 1.67(0.12,3.17) | 1.81(-1.78,6.04) | -0.22(-0.69,0.10) | 0.28(0.06,0.50) | 0.04(0.02,0.06) | 0.10(-0.02,0.26) | 0.02(0.00,0.04) | 2.55(0.01,8.98) | 166.46(34.03,313.70) | 37.78(17.23,61.97) | 44.38(21.11,71.58) | 50.56(-49.60,145.80) | -7.07(-20.32,3.85) | 5.66(1.27,10.15) | 0.73(0.34,1.19) | 2.77(-0.11,6.12) | 0.39(0.11,0.73) | 47.87(0.15,170.22) |

stable4. The Age-standardized rate estimated annual percent change for ischemic stroke attributable to dietary factors during 1990-2021 (A. Death B. DALYs.)

|  | A | | | | | | | | | | B | | | | | | | | | |
| --- | --- | --- | --- | --- | --- | --- | --- | --- | --- | --- | --- | --- | --- | --- | --- | --- | --- | --- | --- | --- |
|  | Dietary risks | Diet low in fruits | Diet low in vegetables | Diet low in whole grains | Diet high in red meat | Diet high in processed meat | Diet high in sugar-sweetened beverages | Diet low in fiber | Diet low in omega-6 polyunsaturated fatty acids | Diet high in sodium | Dietary risks | Diet low in fruits | Diet low in vegetables | Diet low in whole grains | Diet high in red meat | Diet high in processed meat | Diet high in sugar-sweetened beverages | Diet low in fiber | Diet low in omega-6 polyunsaturated fatty acids | Diet high in sodium |
| global | -1.95(-2.05,-1.84) | -2.36(-2.51,-2.21) | -2.78(-2.94,-2.63) | -2.21(-2.36,-2.05) | - | -4.23(-4.56,-3.91) | -1.81(-1.93,-1.68) | -2.87(-3.10,-2.64) | -1.89(-2.00,-1.78) | -1.53(-1.61,-1.44) | -1.70(-1.80,-1.61) | -2.17(-2.30,-2.03) | -2.50(-2.68,-2.32) | -1.80(-1.94,-1.67) | - | -4.00(-4.35,-3.66) | -1.08(-1.21,-0.94) | -2.50(-2.70,-2.30) | -1.64(-1.75,-1.54) | -1.33(-1.41,-1.25) |
| High-middle SDI | -2.42(-2.64,-2.20) | -4.12(-4.52,-3.72) | -4.79(-4.97,-4.60) | -3.09(-3.39,-2.79) | - | -4.99(-5.51,-4.48) | -1.30(-1.42,-1.18) | -4.19(-4.77,-3.61) | -2.63(-2.89,-2.38) | -1.59(-1.76,-1.41) | -2.23(-2.44,-2.03) | -3.94(-4.29,-3.58) | -4.64(-4.89,-4.39) | -2.72(-2.99,-2.44) | - | -5.26(-5.82,-4.69) | -0.68(-0.85,-0.51) | -3.72(-4.25,-3.19) | -2.42(-2.67,-2.17) | -1.39(-1.55,-1.22) |
| High SDI | -3.64(-3.81,-3.47) | -4.21(-4.38,-4.03) | -4.43(-4.64,-4.21) | -3.45(-3.61,-3.28) | 0.94(-2.14,4.11) | -3.75(-3.94,-3.56) | -3.16(-3.40,-2.92) | -4.49(-4.64,-4.33) | -3.93(-4.07,-3.79) | -3.61(-3.77,-3.45) | -2.74(-2.89,-2.60) | -2.95(-3.09,-2.81) | -3.02(-3.24,-2.80) | -2.17(-2.29,-2.04) | -0.10(-0.86,0.67) | -2.65(-2.82,-2.48) | -1.85(-2.05,-1.64) | -3.19(-3.29,-3.08) | -3.24(-3.38,-3.10) | -3.08(-3.24,-2.92) |
| Low-middle SDI | -0.91(-0.97,-0.86) | -1.18(-1.27,-1.10) | -1.85(-1.96,-1.75) | -0.87(-0.91,-0.83) | - | -0.36(-0.41,-0.31) | 1.11(1.01,1.20) | -1.58(-1.70,-1.46) | -0.69(-0.75,-0.64) | -0.54(-0.59,-0.49) | -0.97(-1.02,-0.92) | -1.23(-1.31,-1.16) | -1.87(-1.97,-1.77) | -0.70(-0.74,-0.67) | - | -0.04(-0.08,0.00) | 1.24(1.17,1.31) | -1.60(-1.70,-1.49) | -0.70(-0.75,-0.66) | -0.62(-0.67,-0.56) |
| Low SDI | -0.81(-0.88,-0.74) | -0.65(-0.75,-0.55) | -1.22(-1.31,-1.12) | -0.88(-0.97,-0.79) | - | -0.63(-0.69,-0.57) | 0.14(-0.04,0.31) | -1.22(-1.45,-0.98) | -0.69(-0.76,-0.62) | -0.62(-0.68,-0.57) | -0.87(-0.94,-0.79) | -0.75(-0.86,-0.65) | -1.19(-1.28,-1.10) | -0.85(-0.94,-0.76) | - | -0.60(-0.66,-0.54) | 0.08(-0.10,0.26) | -1.20(-1.44,-0.96) | -0.79(-0.85,-0.72) | -0.70(-0.74,-0.67) |
| Middle SDI | -1.31(-1.37,-1.25) | -2.38(-2.47,-2.29) | -3.86(-4.07,-3.64) | -1.16(-1.26,-1.06) | - | 0.09(-0.02,0.19) | 1.00(0.91,1.09) | -2.66(-2.82,-2.50) | -1.16(-1.26,-1.06) | -1.11(-1.18,-1.03) | -1.23(-1.29,-1.18) | -2.34(-2.44,-2.24) | -3.69(-3.97,-3.42) | -1.01(-1.09,-0.94) | - | 0.45(0.32,0.57) | 1.33(1.20,1.45) | -2.44(-2.56,-2.31) | -1.13(-1.20,-1.06) | -1.03(-1.09,-0.97) |
| Andean Latin America | -2.62(-2.86,-2.37) | -2.74(-3.00,-2.49) | -2.93(-3.14,-2.72) | -3.06(-3.33,-2.79) | - | -1.69(-1.93,-1.44) | -0.91(-1.25,-0.57) | -3.58(-3.79,-3.37) | -2.44(-2.66,-2.23) | -2.40(-2.66,-2.15) | -2.63(-2.88,-2.37) | -2.51(-2.75,-2.28) | -3.07(-3.30,-2.83) | -2.89(-3.14,-2.63) | - | -1.28(-1.57,-0.99) | -0.64(-1.05,-0.23) | -3.43(-3.64,-3.22) | -2.37(-2.58,-2.16) | -2.37(-2.63,-2.11) |
| Australasia | -4.08(-4.23,-3.93) | -4.89(-5.09,-4.70) | -5.03(-5.18,-4.88) | -4.25(-4.41,-4.10) | -5.07(-5.37,-4.77) | -3.05(-3.15,-2.94) | -3.16(-3.35,-2.96) | -5.51(-5.68,-5.34) | -4.19(-4.31,-4.06) | -4.02(-4.16,-3.89) | -3.17(-3.35,-2.98) | -3.52(-3.75,-3.28) | -3.93(-4.17,-3.70) | -2.79(-2.93,-2.64) | -3.13(-3.38,-2.88) | -2.36(-2.50,-2.22) | -2.49(-2.74,-2.24) | -3.95(-4.10,-3.80) | -3.61(-3.76,-3.45) | -3.48(-3.65,-3.30) |
| Caribbean | -1.64(-1.74,-1.54) | -1.59(-1.69,-1.50) | -1.77(-1.91,-1.64) | -1.48(-1.62,-1.34) | - | -0.27(-0.50,-0.04) | 0.88(0.53,1.24) | -2.90(-3.14,-2.66) | -1.69(-1.80,-1.58) | -1.74(-1.82,-1.65) | -1.39(-1.51,-1.27) | -1.36(-1.46,-1.26) | -1.49(-1.65,-1.33) | -1.28(-1.44,-1.13) | - | 0.18(-0.05,0.42) | 1.38(1.03,1.73) | -2.54(-2.80,-2.28) | -1.52(-1.65,-1.39) | -1.43(-1.52,-1.34) |
| Central Asia | -1.92(-2.19,-1.65) | -3.52(-4.03,-3.01) | -5.80(-6.78,-4.81) | -1.35(-1.72,-0.98) | - | -1.10(-1.31,-0.88) | 0.16(-0.20,0.53) | -3.79(-4.64,-2.92) | -1.29(-1.61,-0.96) | -2.07(-2.29,-1.84) | -2.04(-2.32,-1.75) | -3.68(-4.18,-3.18) | -6.56(-7.61,-5.50) | -1.42(-1.77,-1.07) | - | -0.92(-1.12,-0.72) | 0.39(0.04,0.73) | -3.94(-4.77,-3.10) | -1.47(-1.82,-1.12) | -2.34(-2.58,-2.10) |
| Central Europe | -3.03(-3.15,-2.91) | -3.63(-3.85,-3.41) | -5.26(-5.51,-5.01) | -3.46(-3.62,-3.30) | - | -1.85(-1.95,-1.75) | -1.42(-1.52,-1.32) | -3.54(-3.91,-3.18) | -3.16(-3.34,-2.99) | -3.06(-3.19,-2.94) | -2.92(-3.03,-2.81) | -3.29(-3.48,-3.11) | -5.12(-5.35,-4.88) | -3.22(-3.38,-3.07) | - | -1.49(-1.57,-1.40) | -1.05(-1.19,-0.91) | -3.17(-3.50,-2.84) | -3.04(-3.20,-2.88) | -3.04(-3.16,-2.92) |
| Central Latin America | -2.57(-2.80,-2.35) | -2.68(-2.91,-2.45) | -3.37(-3.72,-3.02) | -2.61(-2.89,-2.34) | - | -2.25(-2.42,-2.08) | -2.04(-2.17,-1.91) | -2.80(-3.15,-2.44) | -2.62(-2.81,-2.43) | -2.43(-2.63,-2.23) | -2.46(-2.71,-2.21) | -2.44(-2.68,-2.19) | -3.23(-3.62,-2.85) | -2.37(-2.66,-2.09) | - | -1.99(-2.19,-1.79) | -1.74(-1.90,-1.58) | -2.58(-2.94,-2.22) | -2.48(-2.68,-2.27) | -2.35(-2.56,-2.14) |
| Central sub-Saharan Africa | -0.70(-0.81,-0.60) | 0.15(-0.18,0.48) | -1.17(-1.27,-1.07) | -0.47(-0.62,-0.33) | - | -1.55(-1.73,-1.37) | -2.09(-2.57,-1.61) | 0.83(0.21,1.45) | -0.53(-0.60,-0.47) | -0.12(-0.21,-0.03) | -0.83(-0.93,-0.73) | 0.07(-0.26,0.39) | -1.30(-1.40,-1.19) | -0.55(-0.69,-0.40) | - | -1.66(-1.85,-1.46) | -2.18(-2.69,-1.68) | 0.69(0.09,1.29) | -0.65(-0.71,-0.60) | -0.16(-0.23,-0.09) |
| East Asia | -0.98(-1.14,-0.82) | -3.29(-3.46,-3.12) | -8.97(-9.31,-8.62) | -0.79(-1.04,-0.53) | - | 1.10(0.88,1.33) | 3.70(3.52,3.88) | -3.92(-4.22,-3.62) | -1.09(-1.33,-0.85) | -0.87(-1.05,-0.68) | -0.87(-0.98,-0.77) | -3.35(-3.48,-3.22) | -9.40(-9.87,-8.93) | -0.63(-0.82,-0.44) | - | 1.62(1.40,1.83) | 4.25(4.06,4.45) | -3.54(-3.78,-3.31) | -1.13(-1.30,-0.97) | -0.79(-0.92,-0.65) |
| Eastern Europe | -3.43(-4.00,-2.86) | -4.00(-4.64,-3.34) | -2.14(-2.40,-1.88) | -3.43(-3.99,-2.87) | - | -4.80(-5.57,-4.02) | -0.30(-0.59,-0.01) | -3.53(-4.56,-2.49) | -3.01(-3.56,-2.46) | -2.57(-3.10,-2.04) | -3.30(-3.84,-2.75) | -3.72(-4.32,-3.11) | -1.65(-1.83,-1.46) | -3.13(-3.64,-2.61) | - | -5.24(-6.04,-4.42) | -0.07(-0.37,0.23) | -3.09(-4.09,-2.08) | -2.80(-3.33,-2.27) | -2.36(-2.89,-1.83) |
| Eastern Sub-Saharan Africa | -1.06(-1.13,-0.99) | -0.83(-0.93,-0.73) | -1.39(-1.51,-1.27) | -0.88(-0.94,-0.83) | - | 0.13(0.09,0.18) | 1.21(1.10,1.32) | -2.05(-2.18,-1.92) | -0.68(-0.73,-0.64) | -1.14(-1.21,-1.08) | -1.10(-1.18,-1.03) | -0.94(-1.02,-0.85) | -1.43(-1.54,-1.31) | -0.90(-0.95,-0.84) | - | 0.19(0.14,0.23) | 1.34(1.23,1.45) | -1.88(-2.00,-1.76) | -0.73(-0.78,-0.69) | -1.22(-1.28,-1.16) |
| High-income Asia Pacific | -5.52(-5.74,-5.29) | -5.12(-5.24,-5.01) | -5.38(-5.65,-5.10) | -3.93(-4.11,-3.75) | - | -4.59(-4.85,-4.33) | -4.65(-4.93,-4.38) | -4.20(-4.37,-4.03) | -5.23(-5.41,-5.05) | -5.90(-6.18,-5.62) | -4.45(-4.66,-4.25) | -3.10(-3.17,-3.03) | -4.00(-4.33,-3.67) | -2.29(-2.42,-2.16) | - | -3.47(-3.78,-3.15) | -3.40(-3.69,-3.12) | -2.29(-2.40,-2.17) | -4.35(-4.54,-4.16) | -5.31(-5.60,-5.01) |
| High-income North America | -2.30(-2.67,-1.93) | -2.89(-3.35,-2.42) | -1.08(-1.26,-0.90) | -2.54(-2.97,-2.12) | -2.42(-3.25,-1.58) | -2.81(-3.25,-2.37) | -2.79(-3.30,-2.27) | -3.94(-4.41,-3.47) | -3.36(-3.74,-2.98) | -1.74(-2.05,-1.43) | -1.30(-1.52,-1.08) | -1.67(-1.93,-1.40) | 0.18(0.07,0.28) | -1.30(-1.52,-1.09) | -1.35(-1.62,-1.08) | -1.64(-1.95,-1.32) | -1.45(-1.85,-1.04) | -2.98(-3.24,-2.71) | -2.83(-3.15,-2.51) | -0.82(-1.04,-0.59) |
| North Africa and Middle East | -1.68(-1.75,-1.62) | -2.38(-2.50,-2.25) | -2.94(-3.12,-2.77) | -1.47(-1.51,-1.42) | - | -1.07(-1.12,-1.02) | 0.01(-0.10,0.11) | -2.13(-2.37,-1.89) | -1.56(-1.62,-1.50) | -1.35(-1.40,-1.30) | -1.61(-1.66,-1.56) | -2.15(-2.26,-2.04) | -2.71(-2.89,-2.53) | -1.40(-1.44,-1.37) | - | -0.79(-0.83,-0.75) | 0.35(0.28,0.42) | -1.87(-2.10,-1.65) | -1.55(-1.59,-1.51) | -1.30(-1.34,-1.26) |
| Oceania | -0.98(-1.05,-0.92) | -1.36(-1.39,-1.33) | -0.83(-0.86,-0.80) | -0.71(-0.76,-0.66) | - | -0.76(-0.86,-0.66) | -0.40(-0.55,-0.25) | -3.80(-4.13,-3.47) | -0.55(-0.65,-0.45) | -1.09(-1.18,-0.99) | -0.89(-0.95,-0.84) | -1.35(-1.37,-1.33) | -0.78(-0.81,-0.76) | -0.65(-0.69,-0.61) | - | -0.73(-0.80,-0.65) | -0.34(-0.46,-0.21) | -4.29(-4.66,-3.91) | -0.51(-0.60,-0.43) | -0.97(-1.08,-0.87) |
| South Asia | -0.91(-1.01,-0.82) | -1.11(-1.22,-1.01) | -1.89(-1.99,-1.79) | -0.94(-1.04,-0.85) | - | -0.74(-0.86,-0.62) | 1.14(0.95,1.33) | -1.85(-2.00,-1.71) | -0.91(-1.03,-0.79) | -0.25(-0.33,-0.16) | -0.95(-1.03,-0.87) | -1.11(-1.20,-1.01) | -1.86(-1.94,-1.78) | -0.83(-0.91,-0.75) | - | -0.52(-0.61,-0.44) | 1.07(0.87,1.28) | -1.83(-1.98,-1.69) | -1.00(-1.10,-0.90) | -0.24(-0.30,-0.17) |
| Southeast Asia | -1.14(-1.21,-1.06) | -2.42(-2.49,-2.36) | -2.40(-2.53,-2.27) | -0.53(-0.66,-0.39) | - | -0.11(-0.19,-0.04) | 1.87(1.81,1.94) | -1.24(-1.37,-1.11) | -0.32(-0.48,-0.17) | -0.86(-0.97,-0.76) | -1.29(-1.34,-1.25) | -2.52(-2.63,-2.41) | -2.44(-2.61,-2.27) | -0.49(-0.59,-0.38) | - | 0.23(0.15,0.31) | 2.20(2.13,2.27) | -1.22(-1.31,-1.12) | -0.35(-0.46,-0.24) | -1.00(-1.07,-0.92) |
| Southern Latin America | -3.20(-3.36,-3.03) | -4.09(-4.30,-3.89) | -4.46(-4.72,-4.19) | -3.83(-4.02,-3.65) | -3.24(-3.51,-2.97) | -1.56(-1.73,-1.39) | -1.64(-1.80,-1.47) | -3.67(-3.92,-3.41) | -3.28(-3.44,-3.13) | -3.00(-3.16,-2.85) | -2.95(-3.11,-2.79) | -3.69(-3.90,-3.48) | -4.16(-4.44,-3.87) | -3.41(-3.59,-3.23) | -2.49(-2.69,-2.28) | -1.23(-1.36,-1.10) | -1.29(-1.43,-1.16) | -3.08(-3.33,-2.83) | -3.06(-3.20,-2.92) | -2.78(-2.92,-2.65) |
| Southern sub-Saharan Africa | 0.59(0.06,1.12) | 0.65(0.05,1.26) | 0.33(-0.18,0.85) | 0.72(0.19,1.24) | - | 1.60(1.11,2.09) | 2.40(1.88,2.92) | 1.00(0.46,1.54) | 0.26(-0.20,0.73) | 0.47(0.00,0.95) | 0.21(-0.23,0.65) | 0.21(-0.29,0.72) | -0.03(-0.45,0.39) | 0.30(-0.13,0.74) | - | 1.30(0.89,1.73) | 2.02(1.59,2.45) | 0.56(0.11,1.02) | -0.21(-0.59,0.17) | 0.01(-0.39,0.40) |
| Tropical Latin America | -3.34(-3.47,-3.21) | -4.36(-4.60,-4.12) | -4.82(-5.26,-4.38) | -4.03(-4.26,-3.81) | - | -1.18(-1.43,-0.93) | -0.45(-0.69,-0.21) | -5.09(-5.46,-4.72) | -3.07(-3.21,-2.93) | -3.44(-3.54,-3.34) | -3.30(-3.46,-3.13) | -4.38(-4.69,-4.07) | -4.84(-5.37,-4.32) | -4.03(-4.31,-3.74) | - | -1.02(-1.28,-0.75) | -0.52(-0.72,-0.32) | -5.02(-5.47,-4.57) | -3.02(-3.16,-2.88) | -3.46(-3.59,-3.33) |
| Western Europe | -4.47(-4.66,-4.29) | -5.01(-5.23,-4.80) | -4.91(-5.11,-4.72) | -4.55(-4.74,-4.35) | -4.92(-5.44,-4.41) | -4.24(-4.32,-4.15) | -3.88(-4.00,-3.77) | -4.85(-5.07,-4.63) | -4.67(-4.83,-4.51) | -4.35(-4.56,-4.14) | -3.63(-3.84,-3.43) | -3.78(-4.01,-3.56) | -3.82(-4.03,-3.61) | -3.42(-3.62,-3.22) | -3.43(-3.79,-3.07) | -3.55(-3.63,-3.46) | -3.18(-3.29,-3.06) | -3.61(-3.84,-3.39) | -4.04(-4.22,-3.86) | -3.71(-3.94,-3.48) |
| Western sub-Saharan Africa | -0.53(-0.63,-0.42) | -0.65(-0.75,-0.54) | -1.13(-1.19,-1.07) | -0.90(-1.05,-0.76) | - | 0.36(0.27,0.45) | 3.38(3.04,3.72) | -3.98(-4.31,-3.64) | -0.47(-0.58,-0.36) | 0.18(0.04,0.33) | -0.61(-0.71,-0.52) | -0.68(-0.77,-0.59) | -1.12(-1.17,-1.06) | -0.89(-1.03,-0.75) | - | 0.44(0.35,0.54) | 3.51(3.16,3.85) | -4.01(-4.39,-3.62) | -0.50(-0.62,-0.38) | 0.16(0.01,0.31) |

**SFigure 1: EAPC of ASRs of global burden for ischemic stroke attributable to dietary factors by locations and SDI regions. (A) ASDR (B) Age-standardized DALY rate. DALY = disability adjusted life-year. ASDR = age standardized Death rate. ASRs = age standardized rates.**

**
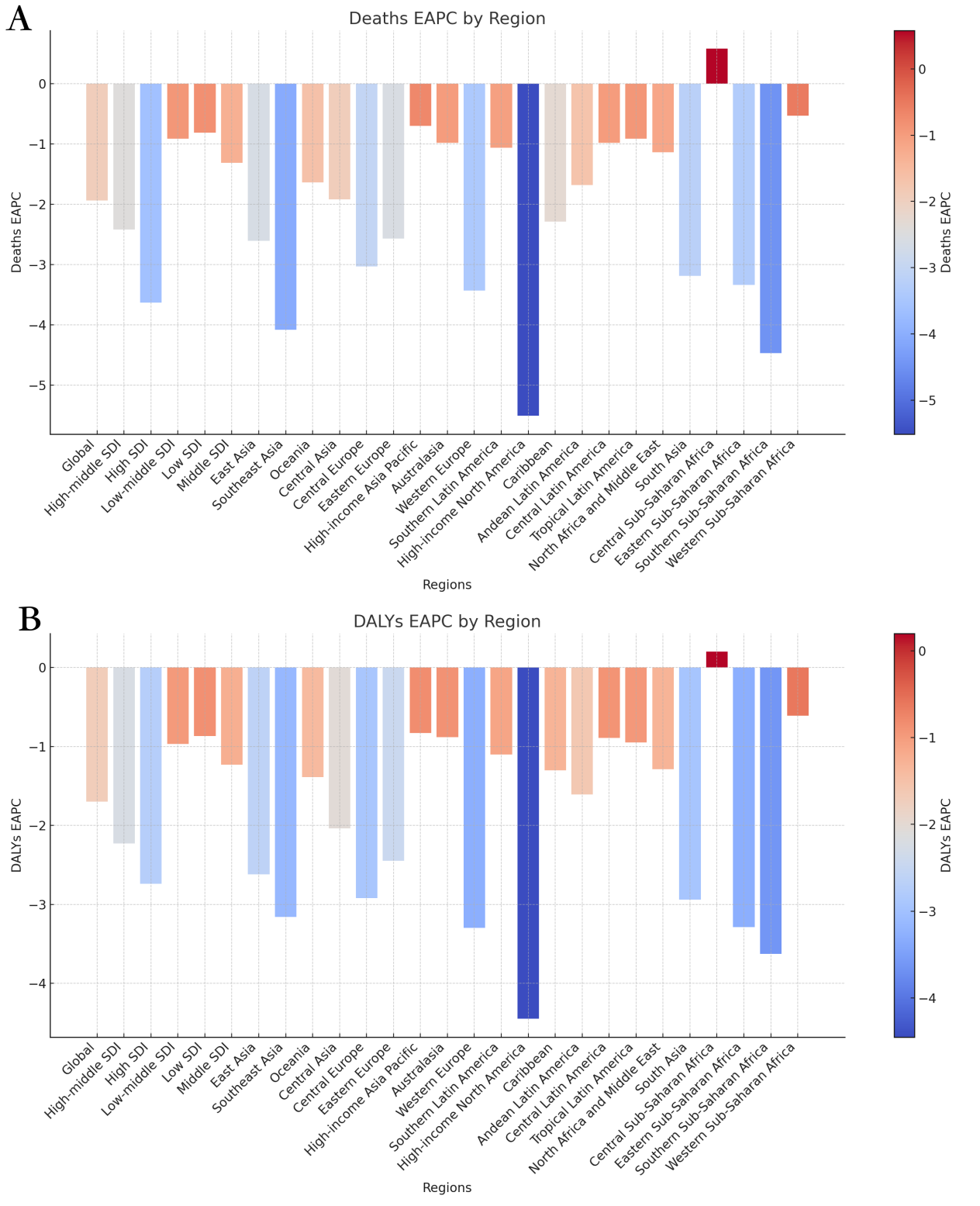
**

**SFigure 2: ASRs of global burden for ischemic stroke attributable to dietary factors in 2021, by locations and SDI regions. (A)ASDR (B) Age-standardized DALY rate. DALY = disability adjusted life-year. ASDR = age standardized Death rate. ASRs = age standardized rates.**

**
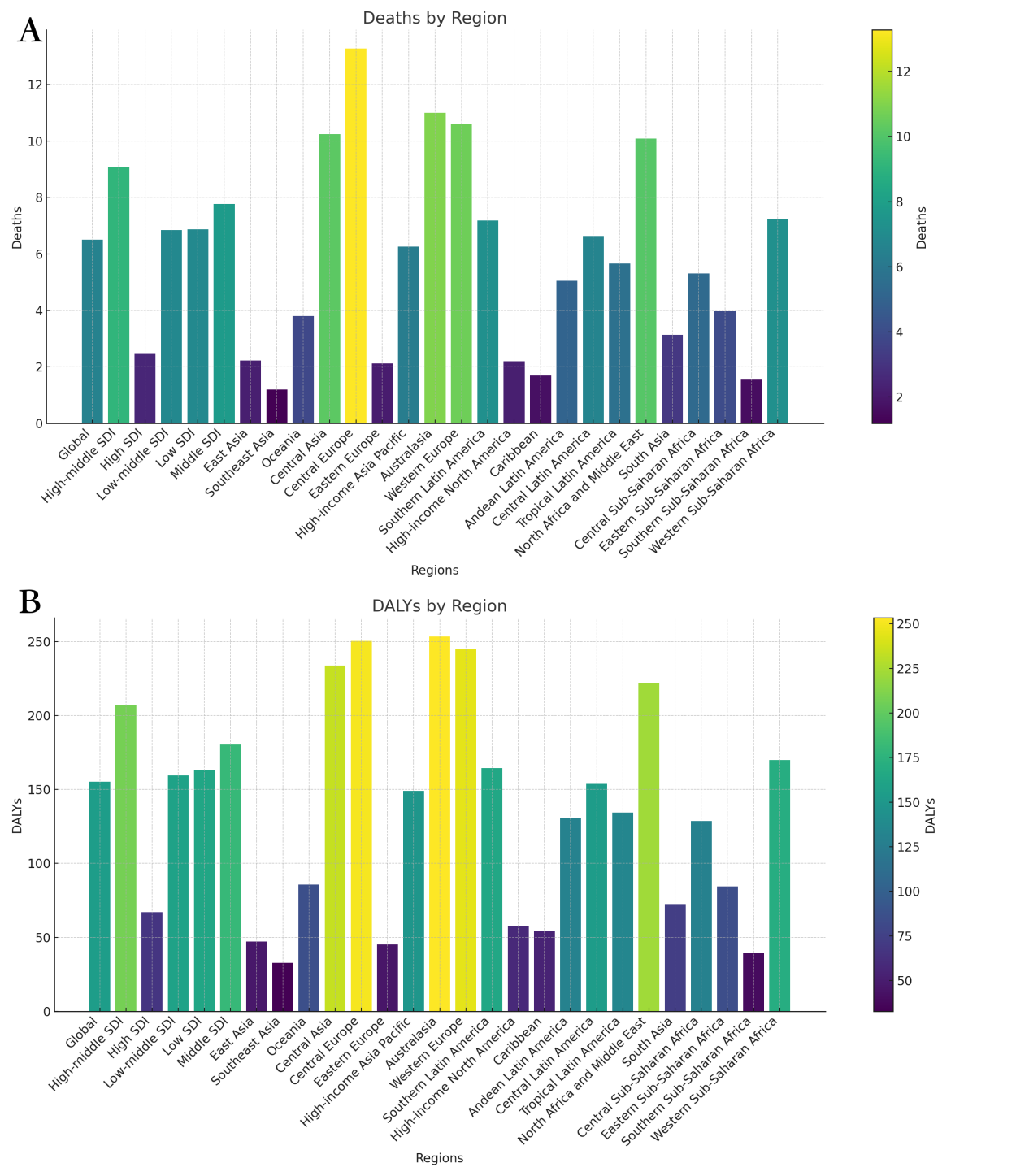
**

**SFigure 3: ASRs of global burden for ischemic stroke attributable to dietary factors in 1990, by regions. (A) ASDR (B) Age-standardized DALY rate. DALY = disability adjusted life-year. ASDR = age standardized Death rate. ASRs = age standardized rates.**

**
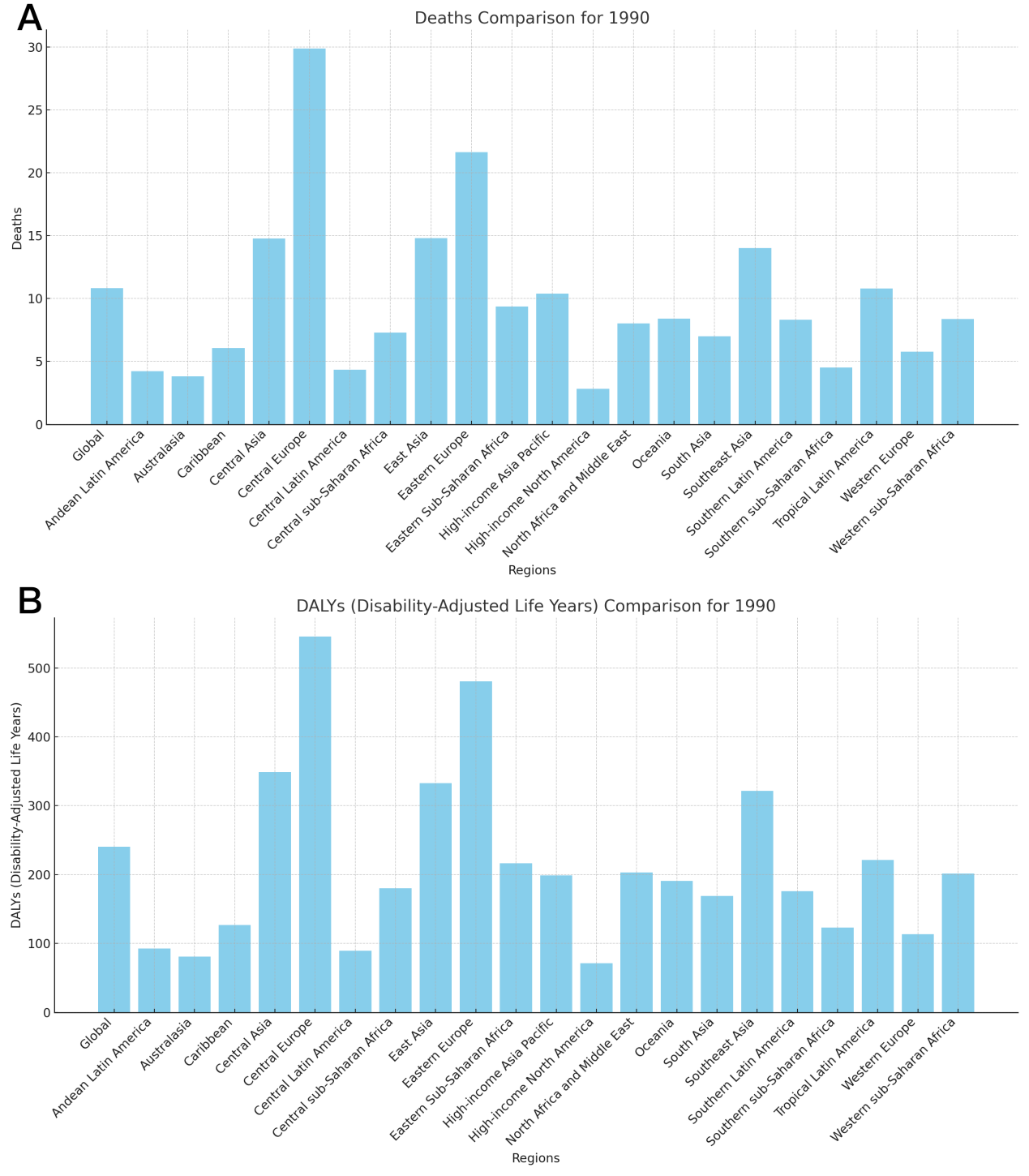
**

**SFigure 4: ASDR of global burden of for ischemic stroke attributable to dietary factors in 204 countries in 2021, by locations.**


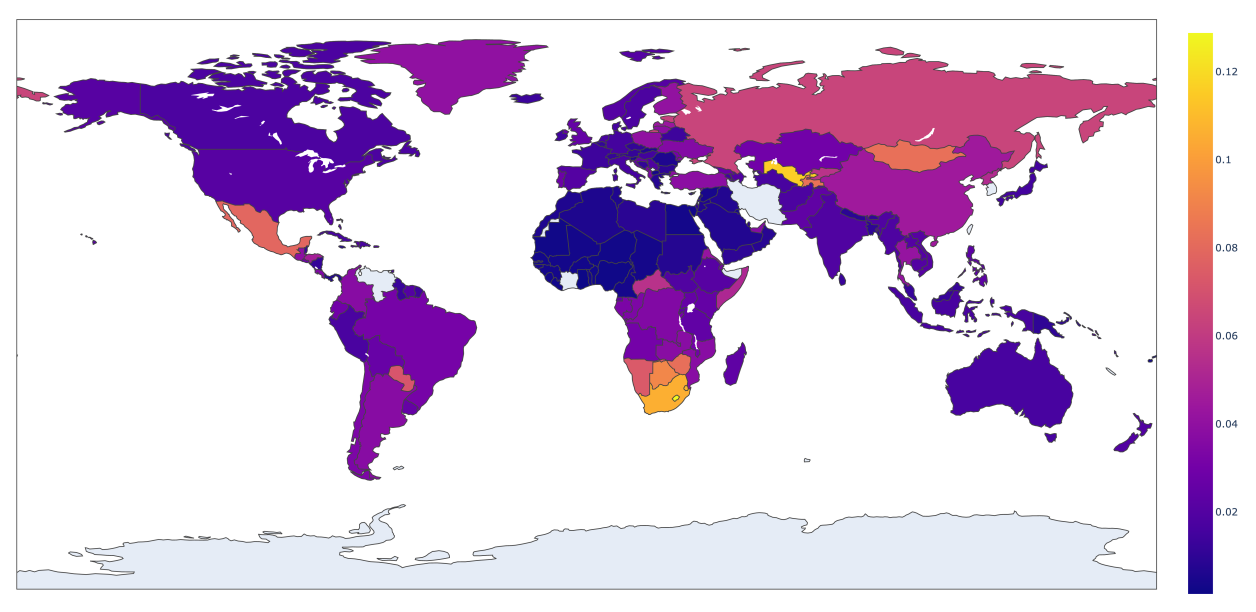


**SFigure 5: Age-standarized DALY rate of global burden of for ischemic stroke attributable to dietary factors in 204 countries in 2021, by locations.**


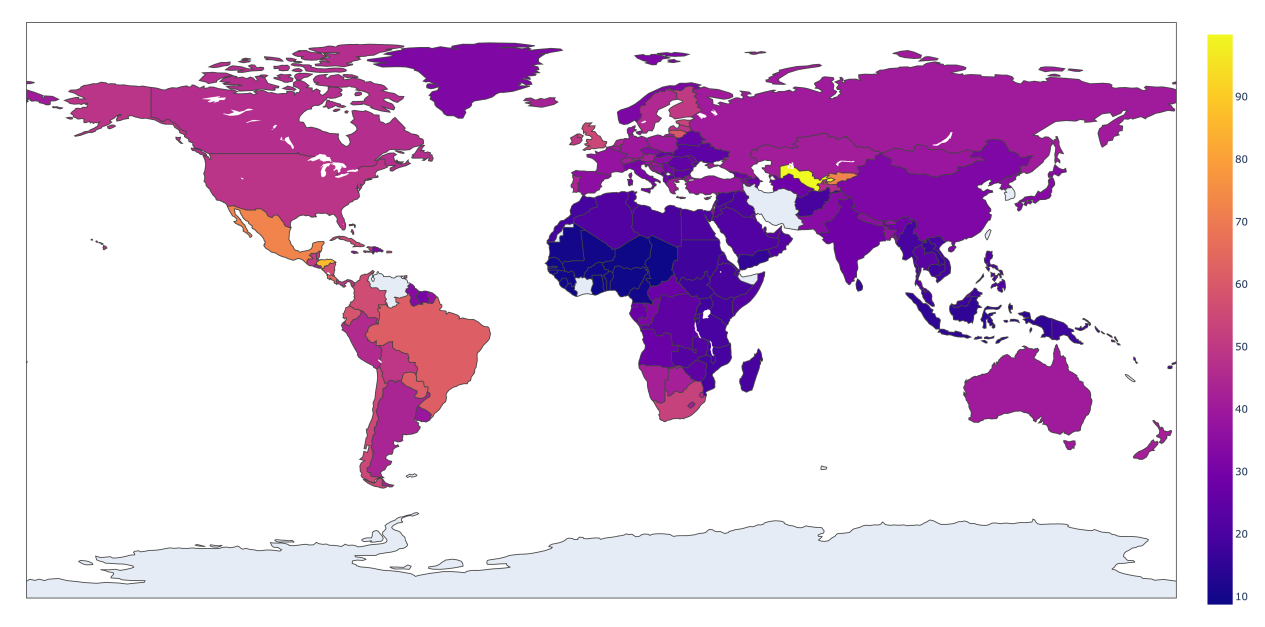

Supplement: Supplementary file 1 [file Table_1.docx]
